# Supplementary material for: How artists experience their own art
Source: PLoS One. 2026 Aug 19;21(8):e0355737. doi: 10.1371/journal.pone.0355737 (PMC13489410; doi:10.1371/journal.pone.0355737)
Supplement: Supplementary file 1 [file pone.0355737.s001.pdf]

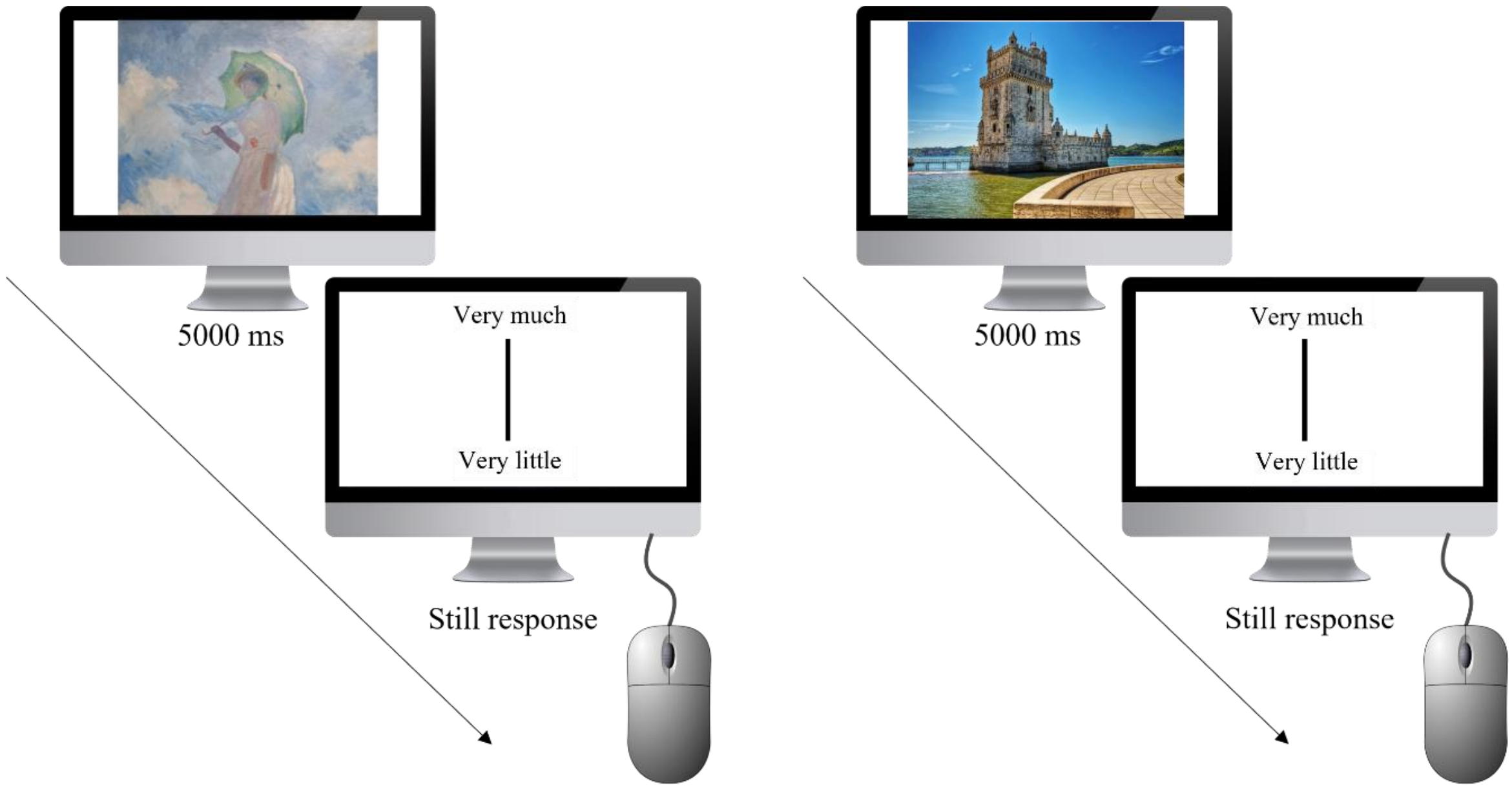

S1 Fig. Trial-timeline of Day1 behavioral tasks. The figure illustrates the trial-time line of the two behavioral judgment tasks performed on Day 1. In the first one, artists provided liking judgments on their own and other artists' paintings (left panel). In the second one, they provided liking judgments on places (right panel).
