# Supplementary material for: How artists experience their own art
Source: PLoS One. 2026 Aug 19;21(8):e0355737. doi: 10.1371/journal.pone.0355737 (PMC13489410; doi:10.1371/journal.pone.0355737)
Supplement: Supplementary file 2 [file pone.0355737.s002.pdf]

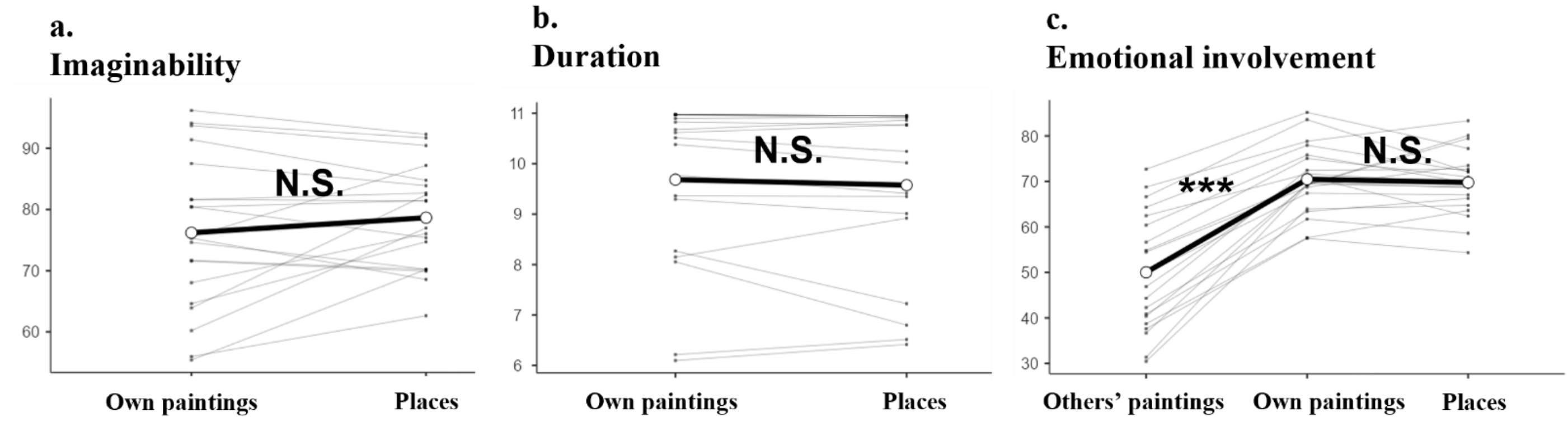

S2 Fig. Behavioral results. The figure shows the results of the mixed models analyses on a) imaginability, b) duration, and c) emotional involvement. Random effects are plotted by subjects with grey lines, while fixed effects are indicated with black lines. *** $p < .001$
