# Supplementary material for: How artists experience their own art
Source: PLoS One. 2026 Aug 19;21(8):e0355737. doi: 10.1371/journal.pone.0355737 (PMC13489410; doi:10.1371/journal.pone.0355737)
Supplement: Supplementary file 3 [file pone.0355737.s003.pdf]

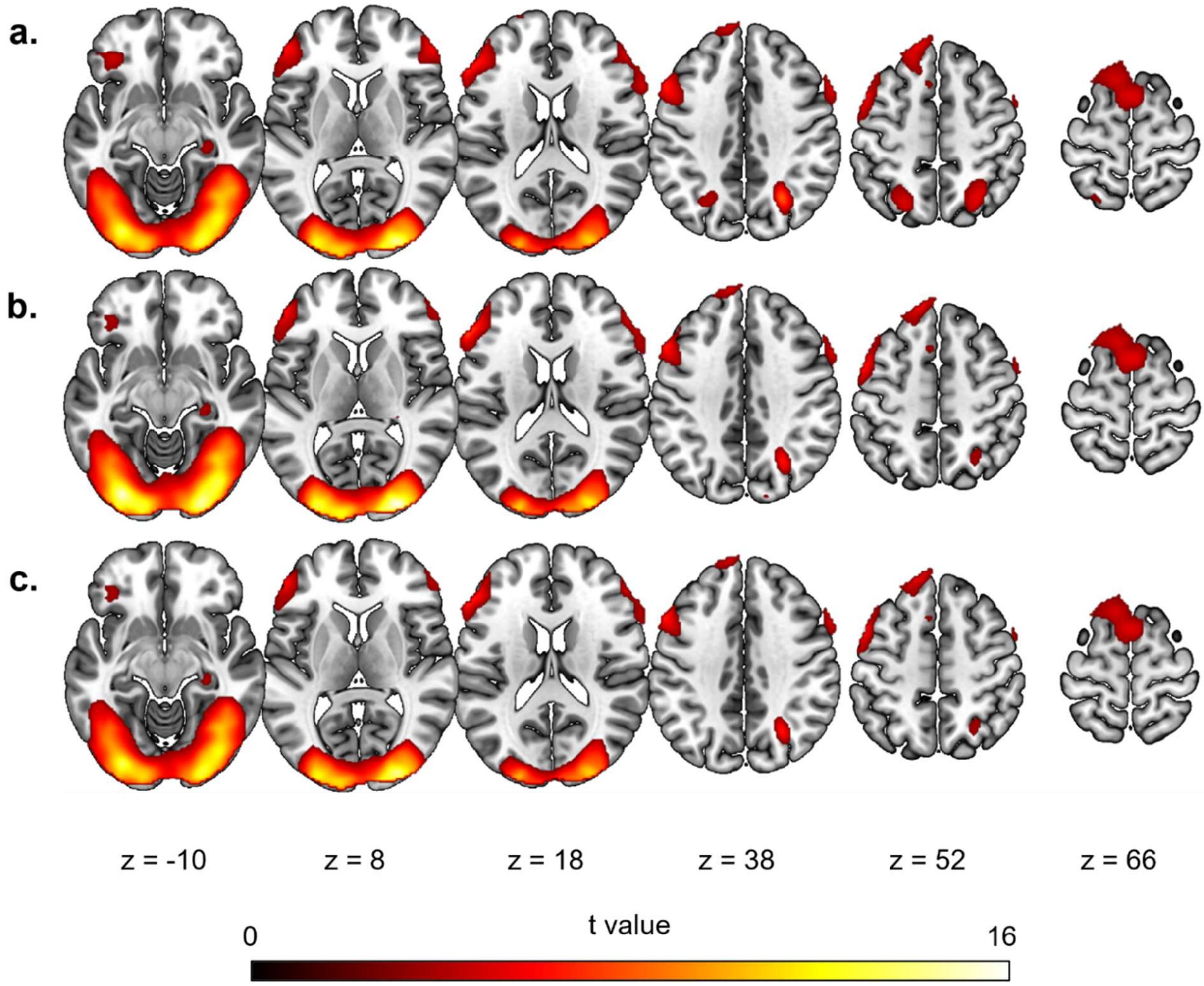

S3 Fig. The neurofunctional underpinnings of aesthetic judgment. The figure illustrates the brain regions involved in a) the simple effect of judging own paintings ("own paintings > implicit baseline"); b) the simple effect of judging others' paintings ("others' paintings > implicit baseline"); and c) their common activations as measured by a conjunction analysis ("own paintings > implicit baseline" ∩ "others' paintings > implicit baseline"). All the data are reported by applying a statistical threshold of $p_{uncorr} < .001$ at the voxel level and $p_{FWER\text{-}corr} < 0.05$ at the cluster level.
