# Supplementary material for: How artists experience their own art
Source: PLoS One. 2026 Aug 19;21(8):e0355737. doi: 10.1371/journal.pone.0355737 (PMC13489410; doi:10.1371/journal.pone.0355737)
Supplement: Supplementary file 4 [file pone.0355737.s004.pdf]

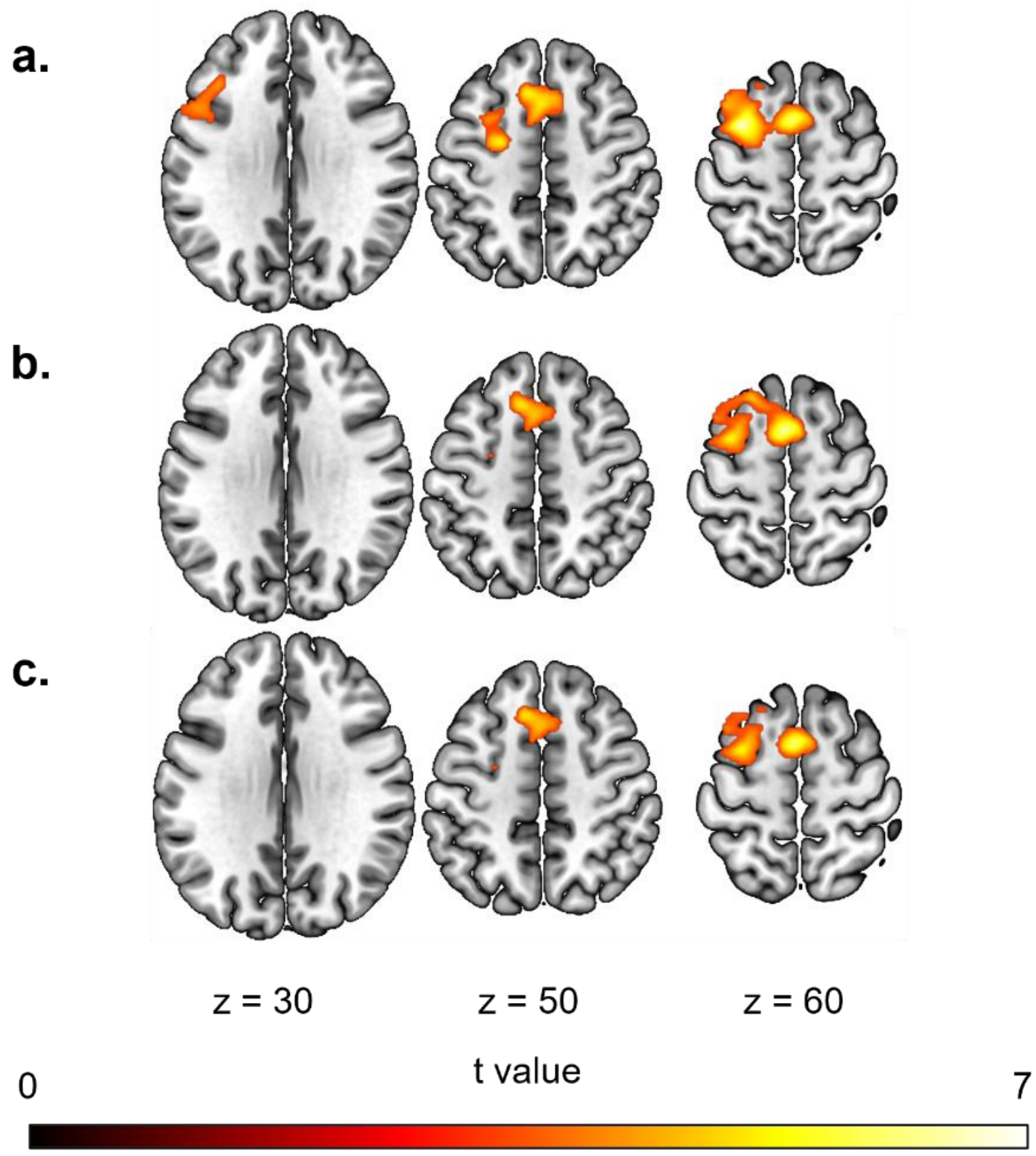

S4 Fig. The neurofunctional underpinnings of reconstruction by imagery. The figure illustrates the brain regions involved in: a) the simple effect of recalling the creative moments ("own paintings > implicit baseline"); b) the simple effect of recalling the visiting of known places ("places > implicit baseline"); and c) their common activations as measured by the conjunction effect ("own paintings > implicit baseline" ∩ "places > implicit baseline"). All the data are reported by applying a statistical threshold of $p_{\text{uncorr}} < .001$ at the voxel level and $p_{\text{FWER-corr}} < 0.05$ at the cluster level.
