# Supplementary material for: How artists experience their own art
Source: PLoS One. 2026 Aug 19;21(8):e0355737. doi: 10.1371/journal.pone.0355737 (PMC13489410; doi:10.1371/journal.pone.0355737)
Supplement: Supplementary file 5 [file pone.0355737.s005.pdf]

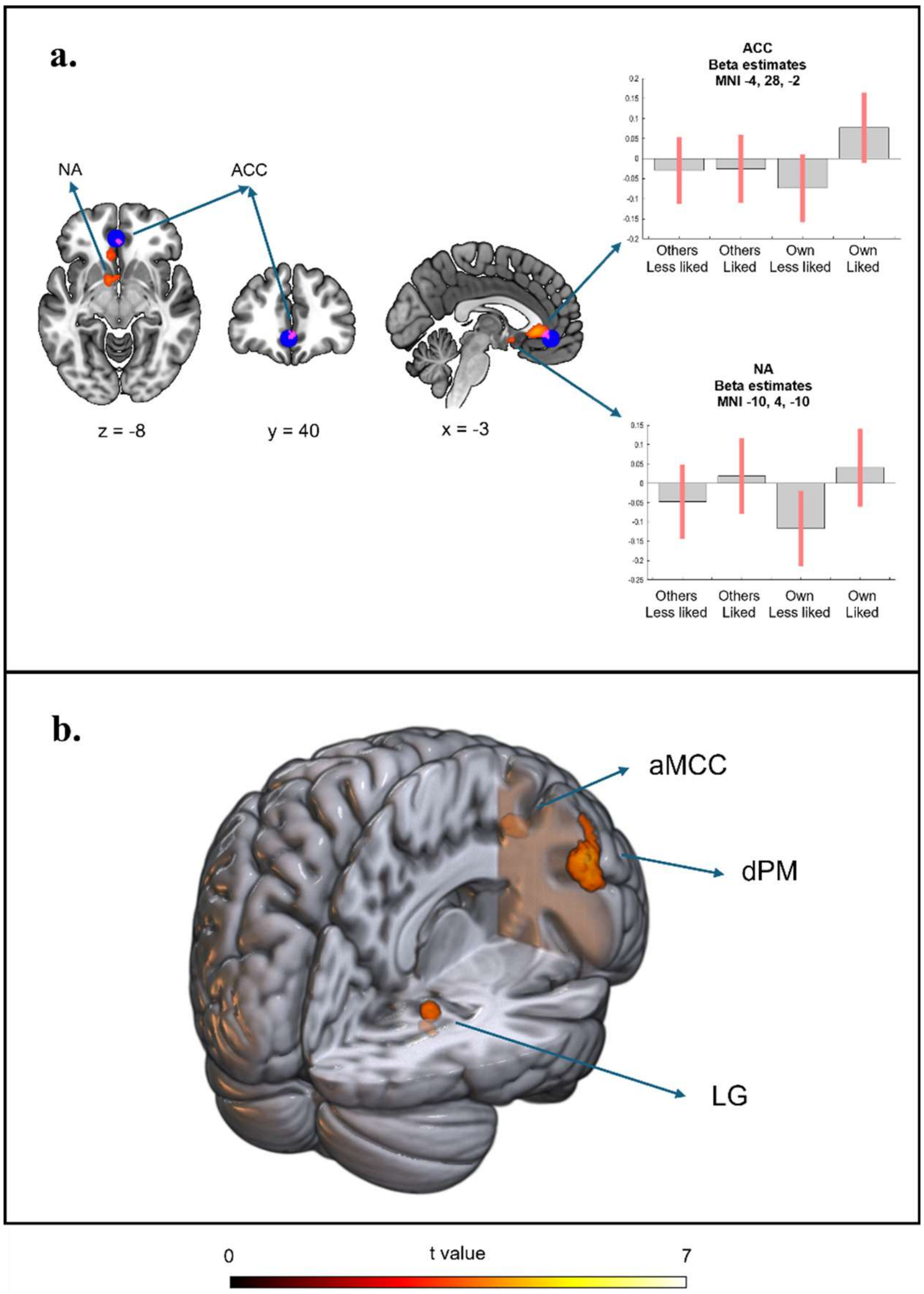

S5 Fig. Extended results of the relationship between Day 1 liking ratings and task-related brain activity. The figure illustrates (a.) the brain regions that show greater engagement during the observation of own artworks that are more appreciated compared with own artworks that are less appreciated (own liked > own less-liked), and their overlap with the field A1 (in blue). The field A1 is a functionally defined region of the medial frontal cortex related to aesthetic experiences of beauty. It is centered at Montreal Neurological Institute (MNI) coordinates -3, 41, –8 mm with a diameter of 15–17 mm. Even though these activations did not survive the correction for multiple comparisons at the whole brain level, the activation within the anterior cingulate cortex survived small volume correction within the field A1. The figure also shows (b.) the brain regions whose activity positively covaried with the mean Day 1 liking ratings for own artworks during the reconstruction by imagery task (as compared with the recall of places). The statistical threshold was set at $p_{uncorr} < .001$ at the voxel level. ACC = anterior cingulate cortex; NA = nucleus accumbens; aMCC = anterior midcingulate; dPM = dorsal premotor cortex; LG = lingual gyrus.
