# Supplementary material for: How artists experience their own art
Source: PLoS One. 2026 Aug 19;21(8):e0355737. doi: 10.1371/journal.pone.0355737 (PMC13489410; doi:10.1371/journal.pone.0355737)
Supplement: Supplementary file 6 [file pone.0355737.s006.pdf]

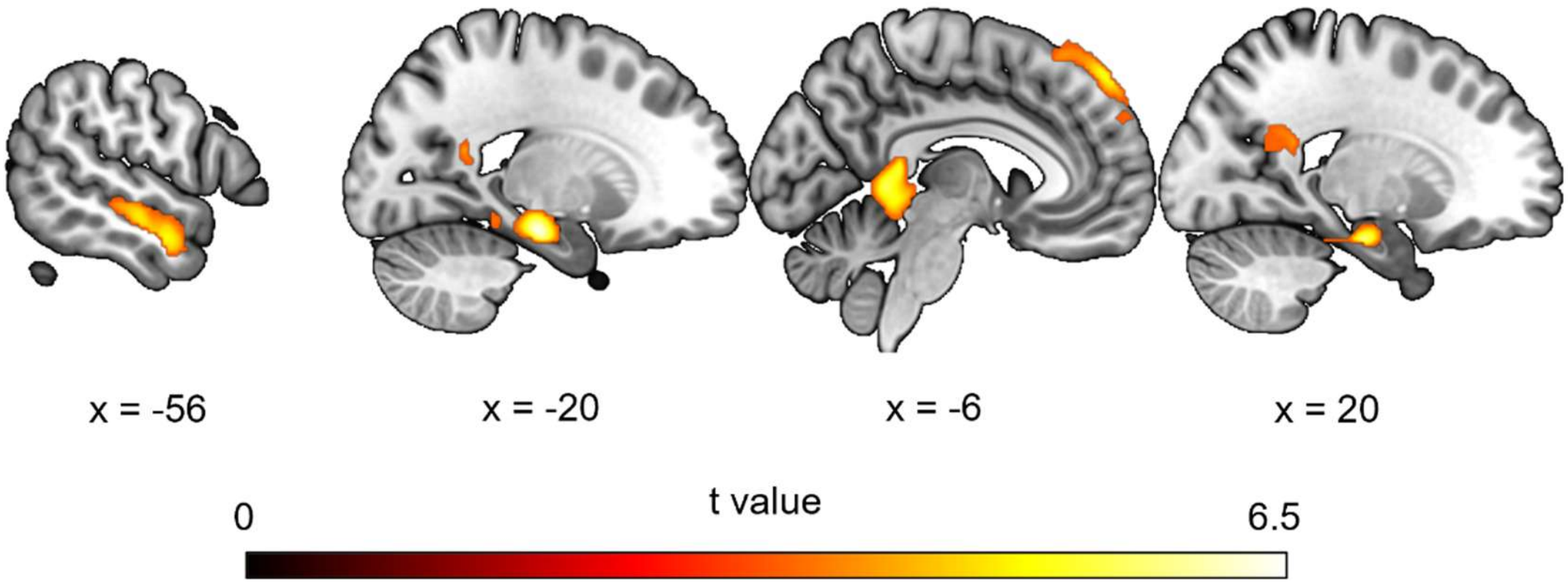

S6 Fig. The specific activations for place reconstruction. The figure shows the brain regions that exhibit a greater involvement during the mental reconstruction of the moments when participants visited the places compared with recalling the creative moments (i.e., the effect "places > own paintings"). The statistical threshold was set at $p_{uncorr} < .001$ at the voxel level and all reported activations survived a family-wise error rate (FWER) correction for multiple comparison at the cluster-level.
