# Supplementary material for: How artists experience their own art
Source: PLoS One. 2026 Aug 19;21(8):e0355737. doi: 10.1371/journal.pone.0355737 (PMC13489410; doi:10.1371/journal.pone.0355737)
Supplement: Supplementary file 7 [file pone.0355737.s007.pdf]

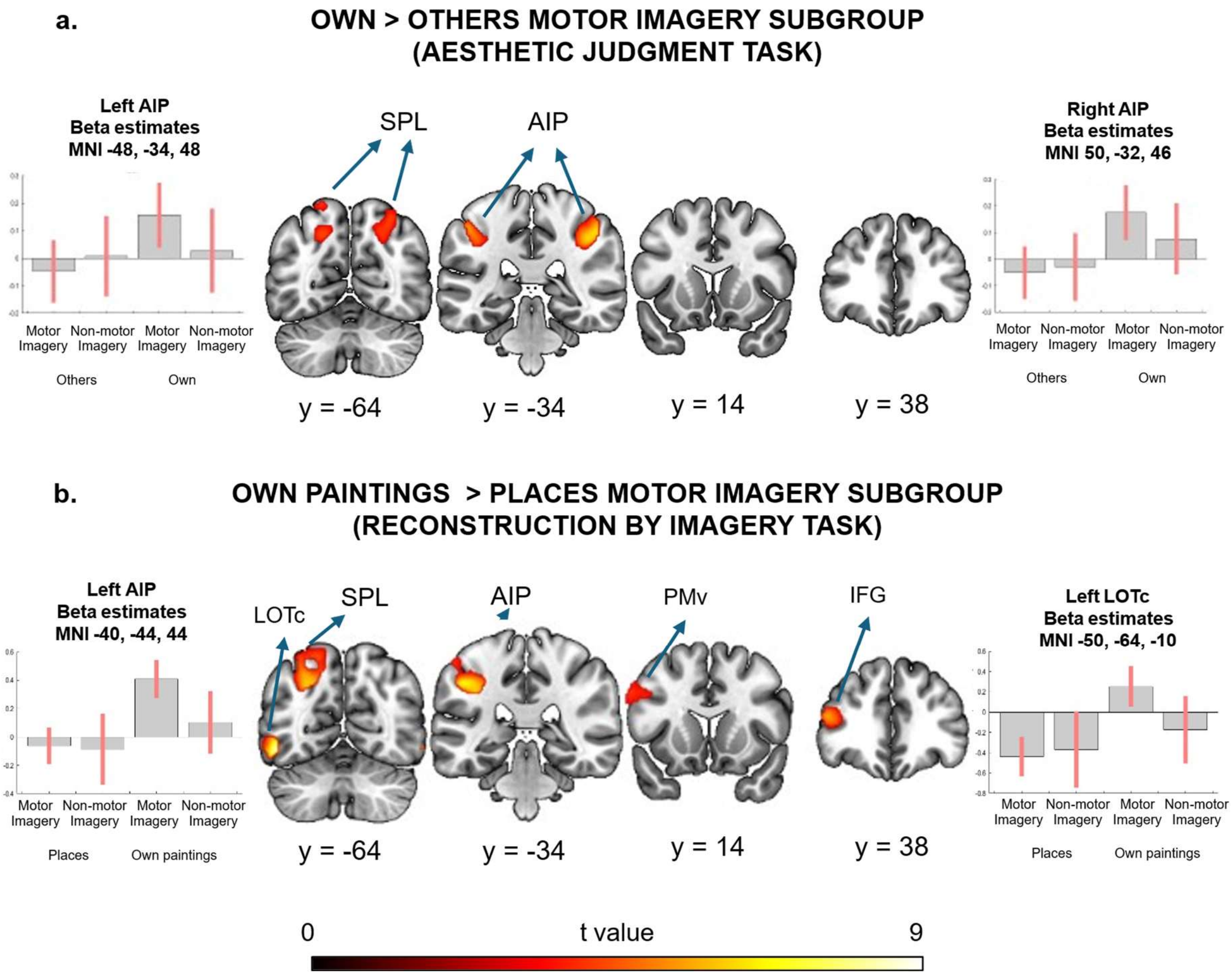

S7 Fig. The neurophysiology of art experience in the motor imagery subgroup. The figure illustrates a) the brain regions that show a greater engagement during the aesthetic judgment of own compared with others' artworks in the motor imagery subgroup, and b) the cerebral regions that exhibit a greater involvement during the reconstruction of the creative moments compared with the control task in the motor imagery subgroup. All the data are reported by applying the same statistical threshold discussed in the text ($p_{\text{uncorr}} < .001$ at the voxel level and $p_{\text{FWER-corr}} < 0.05$ at the cluster or voxel level). For illustrative purposes, we also report the bilateral superior parietal clusters (aesthetic judgment task) though they do not survive the correction for multiple comparisons. LOTc = lateral occipito-temporal cortex; SPL = superior parietal lobule; AIP = anterior intraparietal sulcus; IFG = inferior frontal gyrus; PMv = ventral premotor cortex.
