# Supplementary material for: How artists experience their own art
Source: PLoS One. 2026 Aug 19;21(8):e0355737. doi: 10.1371/journal.pone.0355737 (PMC13489410; doi:10.1371/journal.pone.0355737)
Supplement: Supplementary file 9 [file pone.0355737.s009.pdf]

S2 Table. Neurofunctional results of the simple effect of "others' paintings > implicit baseline" during the aesthetic judgment task.

	Left Hemisphere				Right Hemisphere			
**Brain area (Brodmann area)**	**X**	**Y**	**Z**	**Z-score**	**X**	**Y**	**Z**	**Z-score**
	Left superior and medial frontal cluster $k = 1{,}998$, $p_{FWER\text{-}corr} < 0.001$							
Superior frontal gyrus (8/9)	-12	46	54	5.0*				
	-18	40	56	4.7*				
	-10	62	36	4.6*				
	-18	32	62	4.2				
	-22	26	66	4.1				
Superior medial frontal gyrus (9/10)	-6	64	34	4.7*				
	-8	60	40	4.6*				
	-8	66	30	4.6*				
	-8	56	46	4.5*				
Supplementary motor area (6)	-10	8	78	5.4*				
	0	10	70	4.9*				
Pre-supplementary motor area (6)	-10	22	70	4.4				
	Left middle-inferior frontal cluster $k = 2{,}076$, $p_{FWER\text{-}corr} < 0.001$				*Right middle-inferior frontal cluster* $k = 802$, $p_{FWER\text{-}corr} = 0.007$			
Middle frontal gyrus (9/44/45/46)	-56	20	40	5.5*	52	48	4	4.0
	-50	30	34	4.3	50	46	18	3.4
	-46	24	50	4.0	--	--	--	--
	-42	22	56	3.7	--	--	--	--
	-38	24	58	3.5	--	--	--	--
	-46	32	38	3.4	--	--	--	--
Precentral gyrus (6)	-54	8	48	5.8*	62	14	38	4.7*
	--	--	--	--	60	14	42	4.3
	--	--	--	--	58	8	48	4.2
Inferior frontal gyrus, pars opercularis (44)	--	--	--	--	62	20	34	4.6*
	--	--	--	--	64	18	30	4.5*
	--	--	--	--	60	20	38	4.3
	--	--	--	--	58	24	38	4.0
Inferior frontal gyrus, pars triangularis (44/45)	-60	28	18	6.3*	54	38	20	4.6*
	-52	40	14	5.4*	54	40	16	4.6*
	-56	26	28	4.9*	54	36	24	4.5*
	-56	24	32	4.9*	62	30	18	4.3
	-52	42	0	4.7*	54	44	6	4.0
	--	--	--	--	62	28	22	3.9
	--	--	--	--	62	24	20	3.8
Inferior frontal gyrus, pars orbitalis (47)	-38	34	-12	3.7	--	--	--	--
	Bilateral occipito-temporal cluster $k = 18{,}574$, $p_{FWER\text{-}corr} < 0.001$							
Superior occipital gyrus (7)	--	--	--	--	26	-62	38	5.2*
Middle occipital gyrus (17/18)	-28	-92	2	>8*	--	--	--	--
	-20	-96	-2	>8*	--	--	--	--
	-14	-100	6	7.8*	--	--	--	--
	-24	-96	18	7.4*	--	--	--	--
Inferior occipital gyrus (18/19)	-30	-86	-10	>8*	30	-92	0	>8*
	--	--	--	--	36	-76	-4	>8*
Inferior temporal gyrus (20)	-50	-48	-12	4.6*	--	--	--	--
Fusiform gyrus (37)	-38	-62	-18	6.7*	38	-60	-16	7.7*
	-38	-42	-22	5.5*	38	-44	-18	6.6*
Hippocampus (37)	--	--	--	--	24	-28	0	4.7*

x, y, and z are the stereotactic coordinates of the activations in the Montreal Neurological Institute (MNI) space. The spatial extent of each cluster is reported (k). All reported coordinates are included in clusters that survived a family-wise error rate (FWER) correction for multiple comparison at the cluster-level ($p < .001_{uncorr}$ at the voxel-level) or survived a FWER correction at the voxel-level, as indicated by the asterisks (*).
