# Supplementary material for: How artists experience their own art
Source: PLoS One. 2026 Aug 19;21(8):e0355737. doi: 10.1371/journal.pone.0355737 (PMC13489410; doi:10.1371/journal.pone.0355737)
Supplement: Supplementary file 11 [file pone.0355737.s011.pdf]

S4 Table. Neurofunctional results of the simple effect of "own paintings > implicit baseline" during the reconstruction by imagery task.

Brain area (Brodmann area)	Left Hemisphere X	Y	Z	Z-score	Right Hemisphere X	Y	Z	Z-score
	Bilateral frontal cluster $k = 2{,}182$, $p_{FWER\text{-}corr} < 0.001$							
Superior frontal gyrus (6)	-18	10	70	3.8	--	--	--	--
	-24	-6	52	4.7*				
Supplementary motor area (6)	-4	6	58	5.2*	--	--	--	--
Posterior medial frontal cortex (8/32)	-8	20	52	4.6*	--	--	--	--
	-4	20	46	4.7*	8	22	46	4.4
Middle frontal gyrus (6/8)	-30	4	60	5.5*	--	--	--	--
	-34	16	64	4.5	--	--	--	--
	-30	8	48	3.7	--	--	--	--
	Left premotor cluster $k = 625$, $p_{FWER\text{-}corr} = 0.013$							
Precentral gyrus (6)	-54	8	24	3.7				
	-46	6	44	3.6				
Inferior frontal gyrus, pars opercularis (44)	-50	16	36	4.8*				
Inferior frontal gyrus, pars triangularis (45)	-38	26	30	3.8				
					Right cerebellum $k = 182$, $p_{FWER\text{-}corr} = 0.28$			
Cerebellum					40	-72	-54	4.9*

x, y, and z are the stereotactic coordinates of the activations in the Montreal Neurological Institute (MNI) space. The spatial extent of each cluster is reported (k). All reported coordinates are included in clusters that survived a family-wise error rate (FWER) correction for multiple comparison at the cluster-level ($p < .001_{uncorr}$ at the voxel-level) or survived a FWER correction at the voxel-level, as indicated by the asterisks (*).
