# Supplementary material for: How artists experience their own art
Source: PLoS One. 2026 Aug 19;21(8):e0355737. doi: 10.1371/journal.pone.0355737 (PMC13489410; doi:10.1371/journal.pone.0355737)
Supplement: Supplementary file 13 [file pone.0355737.s013.pdf]

S6 Table. Neurofunctional results of the conjunction analysis of "own paintings > implicit baseline" ∩ "places > implicit baseline" during the reconstruction by imagery task.

	Left Hemisphere				Right Hemisphere			
**Brain area (Brodmann area)**	**X**	**Y**	**Z**	**Z-score**	**X**	**Y**	**Z**	**Z-score**
	Bilateral frontal cluster $k = 1{,}374$, $p_{FWER\text{-}corr} < 0.001$							
Superior frontal gyrus (6)	-18	10	70	3.8	--	--	--	--
Supplementary motor area (6)	-2	8	58	5.2*	--	--	--	--
Posterior medial frontal cortex (8/32)	-8	20	52	4.6*	--	--	--	--
	-2	18	48	4.3	8	20	48	3.9
Middle frontal gyrus (8/9)	-30	4	60	4.6*	--	--	--	--
	-32	16	66	4.0	--	--	--	--
	-38	14	62	3.8	--	--	--	--

x, y, and z are the stereotactic coordinates of the activations in the Montreal Neurological Institute (MNI) space. The spatial extent of each cluster is reported (k). All reported coordinates are included in clusters that survived a family-wise error rate (FWER) correction for multiple comparison at the cluster-level ($p < .001_{uncorr}$ at the voxel-level) or survived a FWER correction at the voxel-level, as indicated by the asterisks (*).
