# Supplementary material for: How artists experience their own art
Source: PLoS One. 2026 Aug 19;21(8):e0355737. doi: 10.1371/journal.pone.0355737 (PMC13489410; doi:10.1371/journal.pone.0355737)
Supplement: Supplementary file 14 [file pone.0355737.s014.pdf]

S7 Table. Results of the control analysis related to the neurofunctional differences between own and others' artworks ("own > others") irrespective of the differences in emotional involvement.

	Left Hemisphere				Right Hemisphere			
**Brain area (Brodmann area)**	**X**	**Y**	**Z**	**Z-score**	**X**	**Y**	**Z**	**Z-score**
					Right Insula# $k = 157$, $p_{FWER\text{-}corr} = 0.317$			
Insula					40	-4	4	3.9#
	Left parietal cluster# $k = 183$, $p_{FWER\text{-}corr} < 0.250$				*Right parietal cluster* $k = 1040$, $p_{FWER\text{-}corr} < 0.001$			
Superior parietal lobule (7)	-26	-70	48	3.3#	34	-60	54	4.1
	-26	-72	56	3.3#	--	--	--	--
	-18	-62	44	3.3#	--	--	--	--
Precuneus (7)	-14	-70	66	3.2#	--	--	--	--
Supramarginal gyrus (40)	--	--	--	--	52	-30	44	4.2
	Left occipito-temporal cluster $k = 273$, $p_{FWER\text{-}corr} = 0.113$							
Inferior temporal gyrus (37)	-50	-60	-6	5.0*				

x, y, and z are the stereotactic coordinates of the activations in the Montreal Neurological Institute (MNI) space. The spatial extent of each cluster is reported (k). Coordinates that survived a FWER correction at the voxel-level are indicated by asterisks (*). We also report the coordinates of the right insular cluster and the left parietal cluster, though they do not survive the correction for multiple comparisons ($p < .001_{uncorr}$ at the voxel-level), as indicated by the hashtag (#).
