# Supplementary material for: How artists experience their own art
Source: PLoS One. 2026 Aug 19;21(8):e0355737. doi: 10.1371/journal.pone.0355737 (PMC13489410; doi:10.1371/journal.pone.0355737)
Supplement: Supplementary file 15 [file pone.0355737.s015.pdf]

S8 Table. Neurofunctional results of the contrast "places > own paintings" during the reconstruction by imagery task.

Brain area (Brodmann area)	Left Hemisphere X	Y	Z	Z-score	Right Hemisphere X	Y	Z	Z-score
	Bilateral medial frontal cluster *k = 652, $p_{FWER\text{-}corr}$ = 0.011*							
Medial superior frontal gyrus (8/9/10)	-6	52	52	4.5	2	56	32	3.4
	-8	34	60	3.6	4	56	36	3.4
	-10	62	30	3.3	--	--	--	--
	-6	60	32	3.2	--	--	--	--
	Left temporal cluster *k = 655, $p_{FWER\text{-}corr}$ = 0.011*				*Right temporal cluster* *k = 229, $p_{FWER\text{-}corr}$ = 0.200*			
Middle temporal gyrus (21)	-62	-6	-16	5.1*	--	--	--	--
	-58	-18	-12	4.3	--	--	--	--
Parahippocampal gyrus (35)	--	--	--	--	20	-16	-22	4.9*
	Bilateral parieto-occipito-temporal cluster *k = 2017, $p_{FWER\text{-}corr}$ < 0.001*							
Precuneus (17/30)	-16	-50	18	3.8	--	--	--	--
	-20	-52	18	3.8	--	--	--	--
Posterior cingulate cortex (29)	-8	-40	8	4.6*	--	--	--	--
Lingual gyrus (27/29)	-8	-46	2	4.7*	8	-48	6	4.6*
	--	--	--	--	10	-40	-2	4.2
Parahippocampal gyrus (35/37)	-20	-20	-18	5.3*	--	--	--	--
	-30	-38	-10	4.1	--	--	--	--
Cerebellum	-12	-36	-14	3.4	--	--	--	--

x, y, and z are the stereotactic coordinates of the activations in the Montreal Neurological Institute (MNI) space. The spatial extent of each cluster is reported (k). All reported coordinates are included in clusters that survived a family-wise error rate (FWER) correction for multiple comparison at the cluster-level ($p < .001_{uncorr}$ at the voxel-level) or survived a FWER correction at the voxel-level, as indicated by the asterisks (*).
