# Supplementary material for: How artists experience their own art
Source: PLoS One. 2026 Aug 19;21(8):e0355737. doi: 10.1371/journal.pone.0355737 (PMC13489410; doi:10.1371/journal.pone.0355737)
Supplement: Supplementary file 16 [file pone.0355737.s016.pdf]

S9 Table. Neurofunctional results of the aesthetic judgment task within the subgroup of artists that employed motor imagery strategies during the reconstruction of art creation ("own > others" masked by the simple effect of own artworks).

	**Left Hemisphere**				**Right Hemisphere**			
**Brain area (Brodmann area)**	**X**	**Y**	**Z**	**Z-score**	**X**	**Y**	**Z**	**Z-score**
	Left superior parietal cluster[#] k = 380, $p_{FWER\text{-}corr}$ = 0.077				*Right superior parietal cluster*[#] k = 373, $p_{FWER\text{-}corr}$ = 0.081			
Superior parietal lobule (7)	-24	-62	68	4.0#	24	-68	50	3.9#
	-20	-62	44	4.0#	30	-64	54	3.8#
	-28	-60	68	4.0#	--	--	--	--
	-36	-58	64	3.9#	--	--	--	--
	-22	-72	54	3.5#	--	--	--	--
	Left inferior parietal cluster k = 235, $p_{FWER\text{-}corr}$ = 0.209				*Right inferior parietal cluster* k = 485, $p_{FWER\text{-}corr}$ = 0.040			
Supramarginal gyrus (2)	--	--	--	--	50	-32	46	5.5*
Inferior parietal lobule (40)	-48	-34	48	4.7*	--	--	--	--

x, y, and z are the stereotactic coordinates of the activations in the Montreal Neurological Institute (MNI) space. The spatial extent of each cluster is reported (k). Reported coordinates are included in clusters that survived a family-wise error rate (FWER) correction for multiple comparison at the cluster-level ($p < .001_{uncorr}$ at the voxel-level) or survived a FWER correction at the voxel-level, as indicated by the asterisks (*). We also report the coordinates of the two superior parietal clusters, though those did not survive the correction for multiple comparisons, as indicated by the hashtag (#).
