# Supplementary material for: How artists experience their own art
Source: PLoS One. 2026 Aug 19;21(8):e0355737. doi: 10.1371/journal.pone.0355737 (PMC13489410; doi:10.1371/journal.pone.0355737)
Supplement: Supplementary file 17 [file pone.0355737.s017.pdf]

S10 Table. **Neurofunctional results of the reconstruction by imagery task within the subgroup of artists that employed motor imagery strategies during the reconstruction of art creation ("own paintings > places" masked by the simple effect of own artworks).**

	Left Hemisphere				Right Hemisphere			
**Brain area (Brodmann area)**	**X**	**Y**	**Z**	**Z-score**	**X**	**Y**	**Z**	**Z-score**
	Left inferior frontal cluster $k = 286$, $p_{FWER\text{-}corr} = 0.129$							
Inferior frontal gyrus, pars triangularis (45)	-48	38	16	4.7*				
	Left ventral premotor cluster $k = 558$, $p_{FWER\text{-}corr} = 0.020$							
Inferior frontal gyrus, pars opercularis (44)	-42	6	24	4.6*				
Precentral gyrus (44)	-60	14	32	3.7				
	Left parieto-occipital cluster $k = 1837$, $p_{FWER\text{-}corr} < 0.001$							
Superior parietal lobule (7)	-22	-64	60	4.2				
	-36	-58	64	3.3				
Inferior parietal lobule (40)	-40	-44	44	6.4*				
	-50	-42	56	4.5				
Middle occipital gyrus (7)	-26	-64	38	5.1*				
	Left occipito-temporal cluster $k = 489$, $p_{FWER\text{-}corr} = 0.031$				*Right occipito-temporal cluster* $k = 6$, $p_{FWER\text{-}corr} = 0.964$			
Inferior temporal gyrus (37)	-50	-64	-10	6.3*	58	-64	-8	4.8*

x, y, and z are the stereotactic coordinates of the activations in the Montreal Neurological Institute (MNI) space. The spatial extent of each cluster is reported (k). Reported coordinates are included in clusters that survived a family-wise error rate (FWER) correction for multiple comparison at the cluster-level ($p < .001_{uncorr}$ at the voxel-level) or survived a FWER correction at the voxel-level, as indicated by the asterisks (*).
