# Supplementary material for: How artists experience their own art
Source: PLoS One. 2026 Aug 19;21(8):e0355737. doi: 10.1371/journal.pone.0355737 (PMC13489410; doi:10.1371/journal.pone.0355737)
Supplement: Supplementary file 18 [file pone.0355737.s018.pdf]

Further fMRI analyses on aesthetic appreciation and artistic production

The effect of motor imagery strategies

We ran additional analyses to explore whether there were neurofunctional dissociations both in the reconstruction of artistic production and the aesthetic appreciation between the subgroup that, during debriefing at the end of the experiment (Day 2), declared to have employed, rather than not, strategies of motor imagery during the reconstruction of art creation. In principle, it is indeed plausible that "motor imagery" artists may exhibit a generally more embodied approach to art than their peers who did not utilize such strategies even when judging works of art. To this end, we replicated the two second-level analyses described in the main text by adding the between-subject factor Group: motor imagery (N =12) vs non-motor imagery (N = 8). This led to two 2x2 ANOVAs having painting (others vs own) as within-subjects factor and group (motor imagery vs non-motor imagery) as between-subjects factor (aesthetic judgment task), or stimuli (places vs own paintings) as within-subjects factor and group (motor imagery vs non-motor imagery) as between-subjects factor (reconstruction by imagery task). For both analyses, we did not find a main effect of group nor an interaction effect. However, we also explored the simple effects "own > others' paintings" (aesthetic judgment task) and "own paintings > places" (reconstruction by imagery task), separately for each group (motor imagery vs. non-motor imagery) to check whether the results replicated those found on the whole sample. Concerning the aesthetic judgment task, the simple effect "own paintings > others' paintings" was masked with the simple effect "own paintings > implicit baseline" (voxel-level $p_{\text{uncorr}} < 0.05$) calculated within each group. Results revealed that the group of artists that employed motor imagery strategies during the reconstruction of artistic creation showed a greater engagement of the anterior intraparietal sulcus bilaterally during the appreciation of own compared with others' paintings. The same trend was observed within the superior parietal lobule bilaterally (k = 380, $p_{\text{uncorr}} = 0.02$ cluster-level; local maximum at MNI -24, -62, 68; z-score = 4.05; $p_{\text{uncorr}} = 0.000026$ voxel-level; k = 373, $p_{\text{uncorr}} = 0.021$ cluster-level; local maximum at MNI 24, -68, 50; z-score = 3.89; $p_{\text{uncorr}} = 0.00005$ voxel-level; S7 Fig, Table S9). The same contrast did not show any significant results in the other group of artists. Concerning the reconstruction by imagery task, the simple effect "own paintings > places" was masked with the simple effect "own paintings > implicit baseline" (voxel-level $p_{uncorr} < 0.05$) calculated within each group. The results showed that artists who employed motor imagery strategies recruited to a greater extent the lateral occipito-temporal cortex bilaterally, the left superior parietal lobule and intraparietal sulcus, the pars triangularis of the inferior frontal gyrus, and the ventral premotor cortex (S7 Fig, Table S10). The same contrast did not show any significant results in the other group of artists, possibly due to the small sample size and the different strategies employed among the artists within this group.
