# Supplementary material for: How artists experience their own art
Source: PLoS One. 2026 Aug 19;21(8):e0355737. doi: 10.1371/journal.pone.0355737 (PMC13489410; doi:10.1371/journal.pone.0355737)
Supplement: Supplementary file 19 [file pone.0355737.s020.pdf]

UNIVERSITA' DEGLI STUDI DI MILANO-BICOCCA
DIPARTIMENTO DI PSICOLOGIA
Piazza dell'Ateneo Nuovo, 1 – 20126 Milano

Milan, 8th of July 2026

To the Academic Editor Federico Giove

Subject: Revision of the Ms. PONE-D-25-42801 to *PLOS ONE*

Dear Editor Federico Giove,

Enclosed, please find the revised version of our manuscript '**How artists experience their own art**', by G. Tomasetig & L.M. Sacheli, M.A. Musco, S. Pizzi, G. Basso, G.F. Spitoni, G. Bottini, L. Pizzamiglio, E. Paulesu.

We would like to thank you for giving us the opportunity to submit a revised version of our manuscript. We are grateful to the Reviewers for their useful comments that we took into the highest consideration. We clarified the critical points raised and, having done so, we believe that the manuscript is now improved.

In the following point-by-point response letter, the Reviewers' comments are reported in **black bold** font; our replies are in blue and preceded by "A.R." (Authors Response). Changes to the main text are highlighted in yellow.

We hope that you and the Reviewers will be satisfied with the revision and that the paper can be accepted for publication in *PLOS ONE*.

If there is anything else you may expect from our side, please do not hesitate to ask at any time.

We look forward to hearing from you.

With kindest regards,

Giulia Tomasetig, Lucia Maria Sacheli, Eraldo Paulesu and Co-Authors

Email: g.tomasetig@campus.unimib.it, lucia.sacheli@unimib.it, eraldo.paulesu@unimib.it

Journal Requirements

1. Please ensure that your manuscript meets PLOS ONE's style requirements, including those for file naming. The PLOS ONE style templates can be found at

https://journals.plos.org/plosone/s/file?id=wjVg/PLOSOne_formatting_sample_main_body.pdf and

https://journals.plos.org/plosone/s/file?id=ba62/PLOSOne_formatting_sample_title_authors_affiliations.pdf

A.R. We thank the Editorial Office for this reminder. We have revised the manuscript to comply with PLOS ONE style requirements. In particular, we have moved the References before the Supporting Information section and checked the structure, file naming, and formatting of the revised submission against the journal templates.

2. Please note that PLOS One has specific guidelines on code sharing for submissions in which author-generated code underpins the findings in the manuscript. In these cases, we expect all author-generated code to be made available without restrictions upon publication of the work. Please review our guidelines at https://journals.plos.org/plosone/s/materials-and-software-sharing#loc-sharing-code and ensure that your code is shared in a way that follows best practice and facilitates reproducibility and reuse.

A.R. We confirm that no author-generated code underpins the findings reported in the manuscript. The analyses were conducted using standard, publicly available software packages described in the Methods section (SPM12 for fMRI preprocessing and analyses; Jamovi/GAMLj for behavioral analyses). Accordingly, there is no custom code to be shared.

3. When completing the data availability statement of the submission form, you indicated that you will make your data available on acceptance. We strongly recommend all authors decide on a data sharing plan before acceptance, as the process can be lengthy and hold up publication timelines. Please note that, though access restrictions are acceptable now, your entire data will need to be made freely accessible if your manuscript is accepted for publication. This policy applies to all data except where public deposition would breach compliance with the protocol approved by your research ethics board. If you are unable to adhere to our open data policy, please kindly revise your statement to explain your reasoning and we will seek the editor's input on an exemption. Please be assured that, once you have provided your new statement, the assessment of your exemption will not hold up the peer review process.

A.R. We confirm that our behavioral and neuroimaging data have already been deposited in the Open Science Framework (OSF) and are currently available for peer review through the following view-only link: https://osf.io/e4fdb/?view_only=7f3dbfdfff70480fb982670c994c01e8. Should the manuscript be accepted, we will make the repository publicly accessible at https://osf.io/e4fdb/.

4. If the reviewer comments include a recommendation to cite specific previously published works, please review and evaluate these publications to determine whether they are relevant and should be cited. There is no requirement to cite these works unless the editor has indicated otherwise.

A.R. We have reviewed and evaluated the publications suggested by the Reviewer. Although we understand that there was no requirement to cite them, we found some of these references useful for providing a more

complete account of previous work on artistic expertise. Accordingly, we have incorporated the relevant references in the revised manuscript where appropriate.

Reviewer:1

Reviewer #1: This is an important study both for the questions asked and the methodology used, namely fMRI. The study investigated brain activity in professional artists with regards to their own artworks and those by others. The results are a contribution to the field of brain and art. The manuscript is well written in its present version and no special revisions are recommended.
A.R. We sincerely thank the Reviewer for their positive evaluation of our work and for recognizing the contribution of the study to the field of cognitive neuroscience of art.

Reviewer:2

Reviewer #2: This manuscript addresses an original and theoretically well-motivated research question, investigating the neurocognitive bases of aesthetic experience and artistic creation in professional painters by contrasting the Embodied Hypothesis with the Perceptual Learning and Inhibitory Hypothesis using fMRI. The experimental design is sophisticated, the stimulus preparation is careful and individualized, and the manuscript is clearly written. Overall, the work has solid scientific merit and makes a meaningful contribution to the emerging field of empirical neuroaesthetics. The revisions requested concern primarily the transparency in the presentation of certain results, a more balanced treatment of the two competing theoretical frameworks, and a more explicit discussion of the methodological limitations inherent to this line of research.
A.R. We thank the Reviewer for their thoughtful and constructive assessment of our manuscript and for recognizing the potential contribution of the study. We have carefully considered the Reviewer's comments, which helped us improve the manuscript considerably. Below, we address each point in detail.

Major Comments
1. Theoretical framing of the hypotheses
I am not entirely convinced that the study hypotheses are formulated in a way that fully respects the theoretical frameworks they aim to test. Based on the original formulation of the Perceptual Learning and Inhibitory Hypothesis, I would expect greater motor inhibition and enhanced perceptual recruitment specifically for paintings that are aesthetically preferred, regardless of whether they are self-authored or made by others, since the theory is fundamentally grounded in the phenomenology of aesthetic pleasure, also with auditory stimuli (Sarasso, P., Barbieri, P., Del Fante, E., Bechis, L., Neppi-Modona, M., Sacco, K., & Ronga, I. (2022). Preferred music listening is associated with perceptual learning enhancement at the expense of self-focused attention. Psychonomic bulletin & review, 29(6), 2108-2121.).
A.R. We thank the Reviewer for this important clarification. We agree that the theoretical framework on aesthetic perception that we refer to most directly predicts enhanced perceptual processing and motor inhibition for aesthetically preferred stimuli, regardless of whether they are self-authored or produced by another artist.

We also agree that this point should have been made more explicit in the previous version of the manuscript. Accordingly, in the revised manuscript, we now distinguish more clearly between two levels of the argument. **First**, we describe the canonical prediction of the Perceptual Learning and Inhibitory Hypothesis in the domain of aesthetic perception, namely that aesthetic pleasure and preferred stimuli should be associated with enhanced perceptual processing and motor inhibition.

Second, we clarify that our prediction regarding self-authored versus other-authored artworks derived from the broader predictive-coding framework within which this hypothesis, at least in the aesthetic perception domain, is situated. More specifically, our reasoning was grounded in predictive-coding and active-inference accounts of aesthetic experience, according to which aesthetic pleasure is closely linked to uncertainty reduction, epistemic value, sense-making, and the progressive construction of a generative model of the artwork (i.e., a model of how the stimulus was generated, of what feelings and intentions occupied the artist's mind when realizing the brushstrokes on the canvas; Van de Cruys, Frascaroli & Friston, 2024). From this perspective, **self-authored works** may entail **lower** epistemic uncertainty for the artist (and, consequently, fewer opportunities for learning) because the artist has privileged access to the generative process that produced the work. By contrast, **works produced by others** may offer greater opportunity for prediction updating and epistemic exploration. We now present this reasoning more explicitly as an inference derived from the broader predictive-coding framework, while also acknowledging that it goes beyond the original theoretical framework's most direct prediction.

In the revised manuscript, we have added/modified the text as follows (pages 5-6, lines 111-134):

"Comparing artists' responses to their own versus others' paintings allowed us to investigate whether the authorship factor modulates the neurofunctional responses during the aesthetic judgment task through embodied simulation mechanisms or through perceptual learning and inhibitory processes. The *Embodied Hypothesis* provides a relatively direct prediction for the self/other contrast. People can recognize and discriminate the products of their past actions from those generated by others [23,24]. This so-called "authorship effect" may be driven by the perception of self-kinematic motor information embedded in static traces, rather than by visual familiarity alone [25,26] or episodic memory [27]. Accordingly, the motor system may resonate more strongly with signals stemming from prior self-produced actions [24,28]. From this perspective, self-generated paintings should elicit motor representations of gestures that artists previously enacted during their creation. Thus, professional artists should show stronger recruitment of brain regions involved in motor resonance while observing their own vs. others' paintings.

By contrast, the *Perceptual Learning and Inhibitory Hypothesis* was originally formulated to explain aesthetic pleasure as emerging from perceptual learning, uncertainty reduction, and "insight" (i.e., Aha experience) on the generative model of the artwork (i.e., a model of the artwork's life cycle, from its conception to its generation [13]). The general prediction from this model is an enhanced perceptual processing and motor inhibition for aesthetically preferred stimuli. However, using the same model, one can make predictions on a self/other authorship manipulation of the stimuli as the one adopted in this experiment. Other-authored artworks may retain greater epistemic uncertainty than self-authored ones, and therefore greater potential for prediction-updating. By contrast, artists have more precise predictions about their own works because they know the actions and intentions that gave rise to them. Consistent with evidence for enhanced prediction and sensory attenuation for self-generated actions [23,28–30], viewing others' paintings should therefore engage perceptual and motor-inhibitory brain regions more strongly, whereas viewing one's own paintings should

place lower demands on perceptual learning. To summarize, what counts here is that the two theories make opposite functional anatomical predictions about the authorship effects".

Furthermore, I could not identify in the original theoretical sources a clear account of the creative process per se within this framework. I would therefore be cautious about advancing directional hypotheses in this regard. This does not diminish the value of the empirical findings: the results can certainly be discussed in light of this theoretical framework, but the authors should be more explicit about the assumptions and potential limitations involved in operationalising these theories within the current experimental design.

A.R. We agree with the Reviewer that the theoretical sources related to aesthetic perception and predictive coding do not provide, by themselves, a direct account of the **creative process** per se. We have therefore revised the manuscript to make the assumptions underlying our theoretical framing more explicit. In our manuscript, the term Perceptual Learning and Inhibitory Hypothesis is used to refer to a broader "Apollonian" position that brings together two distinct but related lines of research: predictive-coding accounts of aesthetic experience, on the one hand, and creative-cognition accounts of artistic production, on the other. These two literatures do not fully belong to the same theoretical framework. In particular, while the literature on aesthetic perception can be situated within predictive-coding and active-inference accounts, the creative-cognition literature does not directly fall within the Predictive Coding framework. However, we considered them together because both emphasize the role of inhibitory processes, although in different domains and with different functional meanings. We have therefore clarified that our theoretical framing does not imply that the creative-cognition literature is part of the Predictive Coding framework. Rather, our proposal reflects a broader attempt to relate perceptual and productive aspects of art experience within the same theoretical space.

The revised Introduction now reads (page 4, lines 77-82):

"Although such Creative Cognition literature does not directly fall within the Predictive Coding framework, it is relevant to the present theoretical contrast because it also emphasizes the role of inhibitory processes, which would be crucial to creating something new. Within this line and following a previous attempt to situate perceptual and productive aspects of art experience within a single theoretical space ([16] see also [17,18]), we refer to this broader "Apollonian" position as the Perceptual Learning and Inhibitory Hypothesis."

We have revised the relevant passage as follows (pages 6-7, lines 135-144):

"The two frameworks also differ in their predictions for the reconstruction by imagery task. On the one hand, according to the Embodied Hypothesis, during the reconstruction of creative moments, professional artists should recruit brain regions involved in sensorimotor simulation to a greater extent than in the control condition. On the other hand, according to the Perceptual Learning and Inhibitory Hypothesis, inhibitory-control processes should play a relevant role in the reconstruction of creative moments. This prediction is grounded in the Creative Cognition literature, where inhibitory control has been proposed to support the suppression of dominant or conventional associations in order to generate novel solutions [14,15]. Thus, in the present study, we used the two tasks to examine whether artists' aesthetic judgment and reconstruction of the creative moment were more strongly characterized by sensorimotor/motor-simulation processes or by perceptual-learning and inhibitory-control mechanisms".

3. Clusters not surviving correction for multiple comparisons

I agree that also non significant results has their value (Chen, G., Taylor, P. A., & Cox, R. W. (2017). Is the statistic value all we should care about in neuroimaging?. NeuroImage, 147, 952-959.). However, several key results discussed in the Main Text and illustrated in Figure 2 do not reach significance after FWER correction. Although the authors flag these with footnotes, they receive equal narrative emphasis throughout the text and are displayed in the main figures without sufficiently prominent caveats. I recommend that the Discussion explicitly distinguish between statistically supported findings and exploratory trends, and that the main conclusions be anchored accordingly.

A.R. We thank the Reviewer for this important point. We agree that, in the previous version of the manuscript, statistically supported effects and substantial trends were not always sufficiently distinguished.

With regard to the contrast "own liked > own less-liked", we acknowledge that this effect did not survive whole-brain FWER correction. However, it should be noted that the crucial trend maps in a region that was highly anticipated based on the literature, namely the so-called "field A1" described by Ishizu & Zeki (2011). Given the functional relevance of this independently defined region for aesthetic experience of beauty, we performed an additional small volume correction on the very region identified by Ishizu & Zeki. Within this search volume, the effect survived an FWER correction. We now report this result explicitly as a small-volume-corrected finding, clearly distinguishing it from effects that survived correction at the whole-brain level.

We have now made this explicit in the Results section (pages 19-20, lines 454-471), which reads:

"We then examined the simple effects of liked > less-liked paintings separately for self-authored and other-authored artworks. For other-authored paintings, no liking-related anatomical effect was present. On the other hand, for self-authored paintings, the contrast own liked > own less-liked revealed a trend in the ventral anterior cingulate cortex (vACC, k = 306, puncorr = 0.044 cluster-level. Local maxima: MNI -4, 28, -2; z-score = 4.28; puncorr = 0.000000009 at the voxel-level, and MNI 0, 38, -8; z-score = 3.39; puncorr = 0.000347 voxel-level; see S5 Fig), within a medial frontal region compatible with the so-called field A1, previously associated with the experience of beauty [35–37]. This activation did not survive whole-brain FWER correction. However, given the functional relevance of field A1 for aesthetic experience of beauty [35–37], we applied a small volume correction (SVC) using an independently defined spherical region of interest centered on the coordinates reported by Ishizu and Zeki [35] for field A1 (MNI: -3, 41, -8; radius: 8 mm). Within this search volume, the effect survived FWER correction (cluster size: k = 34 voxels; $p_{FWER\text{-}SVC} = 0.009$ at the cluster level; local maximum: MNI: 0, 36, -4; z-score = 3.53; $p_{FWER\text{-}SVC} = 0.005$ at the voxel level. Additional local maxima within the same cluster were observed at MNI: -4, 34, -6; z-score = 3.46; $p_{FWER\text{-}SVC} = 0.006$ at the voxel level and MNI: 0, 38, -8; z-score = 3.39; $p_{FWER\text{-}SVC} = 0.007$ at the voxel level; see Fig. 3a). We also found a trend within the left nucleus accumbens (k = 95, puncorr = 0.239 cluster-level. Local maximum: MNI -10, 4, -10; z-score = 3.92; $p_{uncorr} = 0.00004$ voxel-level; see S5 Fig), but this effect did not survive correction for multiple comparisons at the whole brain level, nor did it belong to the volume of interest identified by Ishizu and Zeki (2011) and is therefore reported only as an exploratory observation."

In any case, we removed the uncorrected liking-related results from Figure 2. These results are now presented in the Supporting Information (S5 Fig). Importantly, we also added a new main-text figure, Figure 3, specifically devoted to the liking-related analyses. Unlike S5 Fig, Figure 3 reports only effects that survived

correction for multiple comparisons, either after small volume correction within the independently defined field A1 (aesthetic judgment task, Fig. 3a), or at the whole-brain level (reconstruction by imagery task, Fig. 3b).

We have also revised the Discussion to state explicitly that the liking-related modulation for the contrast own liked > own less-liked survives small volume correction in field A1, while distinguishing this result from effects surviving whole-brain correction. More generally, throughout the Discussion, we now specify which findings are supported by whole-brain correction, which are supported by small volume correction, and which should instead be considered exploratory trends.

The Discussion section (pages 27-28, lines 630-641) now reads:
"Finally, we explored whether the neural activity was modulated by the extent of appreciation of artists' own and others' paintings. We did not find a main effect of liking. However, we identified a trend within the ventral anterior cingulate cortex and the left nucleus accumbens: these regions showed differential activations, based on liking judgments, only when artists observed their artworks, with deactivation when they evaluated their own less-liked paintings. Although these effects did not survive FWER correction at the whole-brain level, the activation within the anterior cingulate cortex survived small volume correction within the so-called field A1, a medial frontal region previously associated with the experience of beauty across the visual and musical domains [35–37]. We also observed a cluster in the left nucleus accumbens, a key node of the mesolimbic reward circuit, which is reliably and directly linked to the experience of pleasure, including in the music domain [51–54]. However, this latter effect did not survive any correction for multiple comparisons and should therefore be considered as exploratory. By contrast, no liking-related response was present during the observation of others' artworks."

4. MRI data preprocessing

(Line 281) The data matrix was interpolated to produce voxels with 2 × 2 × 2 mm dimensions. What was the native acquisition? (4 × 3 × 3 mm?). It should be explicitly acknowledged that upsampling artificially estimates signal values between measured points and may inflate the apparent spatial extent of activation clusters. This limitation should be acknowledged in the Preprocessing section.

A.R. We thank the reviewer for pointing out this omission. The native acquisition resolution was 3×3×4 (x,y,z) mm. As suggested, we have explicitly reported this information in the revised Preprocessing section. We have also added an acknowledgement regarding the limitations of upsampling, specifically noting that it estimates signal values between measured points and may inflate the apparent spatial extent of activation clusters. However, because the same preprocessing procedure was applied to all functional images and all experimental conditions, this step is unlikely to introduce a condition-specific bias in the direct contrasts reported in the manuscript.

The revised text now reads (pages 13-14, lines 314-318): "The native acquisition resolution was 3×3×4 (x,y,z) mm, and the data matrix was then interpolated and upsampled to produce voxels with 2 × 2 × 2 mm dimensions. Because this procedure estimates signal values between originally acquired voxels, it may affect the apparent spatial extent of activation clusters. However, since the same preprocessing procedure was applied to all functional images and experimental conditions, upsampling was unlikely to introduce a condition-specific bias in direct contrasts."

5. Aesthetic appreciation independently of authorship

(Line 501) A question that arises from the current design is whether the authors examined the neural correlates of aesthetic appreciation independently of authorship collapsing across self-authored and others' paintings to identify brain regions that track aesthetic liking regardless of who produced the artwork. It should also be noted that what was measured in the scanner during the appreciation task was an aesthetic judgment, not aesthetic appreciation per se. It would be informative to know whether neural responses differ as a function of liked versus less-liked paintings across both categories. In this light, I would suggest either modeling four regressors of interest (own liked, own less-liked, others' liked, others' less-liked) or, alternatively, correlating the Day 1 liking ratings with the beta estimates from the own > others contrast. What the current data most compellingly support is the statement at line 526 that observing their own paintings automatically triggers internal mental representations of the actions previously performed, while conclusion on aesthetic pleasure would be strengthened by the analyses suggested above.

A.R. We thank the Reviewer for this helpful suggestion. We agree that the task performed in the scanner is more accurately described as an aesthetic judgment task rather than an aesthetic appreciation task. We have therefore changed the name of the task from "aesthetic appreciation task" to "aesthetic judgment task" throughout the revised manuscript.

Regarding the analysis of aesthetic liking independently of authorship, in the previous version of the manuscript, we had already modelled four regressors of interest at the first level: own liked, own less-liked, others' liked, and others' less-liked paintings. These effects were then entered into a further fMRI second-level 2 x 2 ANOVA with painting (own, others) and liking (liked, less-liked) as within-subject factors to examine whether aesthetic judgments (liking) modulated neural responses. However, we acknowledge that, in our previous version of the manuscript, we did not report the main effect of liking collapsing across authorship. In the revised manuscript, in addition to reporting the simple effects of liked > less-liked separately for each stimulus category, namely self-authored and other-authored paintings, we now also report the main effect of liking across both categories, which, however, did not reveal any cluster surviving correction for multiple comparisons.

We have now made this explicit in the Results section (page 19, lines 452-454), which reads:

"Moreover, we were interested in evaluating whether the brains of our artists reacted differently to artworks they liked more. When collapsing across one's own and others' paintings, neither the main effect of liking nor the painting-by-liking interaction effect yielded clusters that survived correction for multiple comparisons."

6. Motor versus visual familiarity

(Lines 538–540 and 547–552) The authors draw on the Calvo-Merino studies to argue that differential motor activations for self-authored versus others' paintings reflect motor rather than visual familiarity. However, the claim at lines 538–540 that this inference "is supported by the distribution of activations" involves circular reasoning: the distribution of activations is precisely what needs to be explained, and invoking it as supporting evidence assumes the conclusion. The activation pattern is equally consistent with the interpretation that motor-like responses reflect motor familiarity specifically, which is in fact

the most parsimonious account, but this cannot be dissociated from visual familiarity on the basis of the current data alone. The ideal design to address this question would have included art historians or art critics (i.e. individuals with extensive visual familiarity with the stimuli but no motor experience in producing them) to determine whether motor-like activations arise from visual familiarity alone or require prior motor engagement.

A.R. We thank the Reviewer for this careful and convincing critique. We agree that the previous wording risked circular reasoning and overstated what can be inferred from the current design. We have removed the sentence claiming that the distribution of activations supports a motor-rather-than-visual familiarity interpretation. In addition, in the Conclusions, we report as an interesting future direction the possibility of studying art historians and critics. This would allow future research to test the two alternative hypotheses in a sample of individuals who have extensive visual familiarity and aesthetic expertise with artworks, but do not possess the motor repertoire associated with artistic production.

Furthermore, the authors state at line 102 that they ensured a high degree of familiarity with others' paintings. It is unclear how this was formally assessed.

A.R. We thank the Reviewer for noting this issue. The previous statement was based on informal comments provided by several artists after the Day 1 behavioral session, who reported knowing most of the other artists' pieces of art. However, familiarity with each other-authored painting was not formally assessed. This has been clarified in the method section (page 9, lines 198-199).

7. Discussion (Lines 563 ff. — Reconstruction by imagery task) In my view, this section presents the most compelling and original contribution of the entire manuscript. The finding that mental reconstruction of the creative process recruits a fronto-parietal network associated with motor cognition provides the most innovative evidence in favor of an embodied account of artistic experience, particularly with respect to creativity. I would encourage the authors to give this result greater prominence in the Abstract and conclusions.

A.R. We are grateful to the Reviewer for this encouraging comment. We have revised the manuscript to give greater prominence to the finding that mentally reconstructing the creative process recruits a fronto-parietal network associated with motor cognition.

The revised Abstract now states: "The reconstruction of their own artistic creation (compared with episodic memory retrieval connected with places) involved the left fronto-parietal network associated with motor cognition, providing evidence that the sensorimotor dimension may be intrinsically entailed in the creative process. Moreover, the aesthetic judgments attributed to one's own artworks positively covaried with activity in the right dorsal premotor cortex during creative reconstruction, suggesting that aesthetic value may be linked to the reactivation of sensorimotor components associated with the creative gesture. Altogether, these results hint that the mental representations of the actions involved in creating art are integral to the overall artistic experience of painters, from conception to appreciation, supporting an embodied view of the artists' experience of art".

As a constructive suggestion, I recommend correlating the individual beta estimates from the regions identified in this contrast with the aesthetic liking ratings collected on Day 1. Such an analysis could reveal whether the degree of motor recruitment during creative reconstruction is modulated by how

much the artists valued their own works aesthetically, a theoretically meaningful finding that would add an important individual-differences dimension to the current results.

A.R. We thank the Reviewer for this valuable suggestion. Following this recommendation, we tested whether individual differences in Day 1 liking ratings for the artists' own works were associated with neural activity during the reconstruction of the creative process. Specifically, we entered the individual contrast images for own paintings > places into a second-level regression analysis and included the mean Day 1 liking score for each artist's own paintings as a covariate.

We specified this in the methods section (pages 16-17, lines 384-395), which now reads:

"We conducted two further fMRI analyses to examine whether aesthetic judgments (liking ratings) provided on Day 1 modulated neural responses during the two tasks (i.e., aesthetic judgment task and reconstruction by imagery task). For the aesthetic judgment run, we separately modelled the own and others' artworks that obtained the best and the worst judgments on Day 1 (own less-liked mean: 43.92 ± 18.14; others' less-liked mean: 43.95 ± 18.23; own liked mean: 83.43 ± 9.63; others' liked mean: 83.42 ± 9.75). Thus, we entered liking (less-liked/liked) as a further within-subjects factor in the fMRI analysis. We modelled four regressors of interest and the corresponding effects at the first level of analysis (own less-liked, own liked, others less-liked, others liked). The four effects were entered into a second-level full-factorial analysis of variance (ANOVA) that included paintings (own vs others) and liking (less-liked vs liked) as within-subject factors. For the reconstruction by imagery run, we applied a weight of +1 to the regressor of own paintings, and a weight of -1 to the regressor of places. We entered the individual contrast images of own paintings > places into a second-level regression analysis and included the mean Day 1 liking score for each artist's own paintings as a covariate."

This analysis revealed a positive association in the right dorsal premotor cortex, together with trends within the anterior midcingulate and the right lingual gyrus. Thus, artists who assigned higher aesthetic value to their own works showed stronger recruitment of a region involved in action preparation, motor organization, and motor representation during the reconstruction of the creative process. We have added this result to the manuscript.

The revised Results section now states (page 20, lines 486-491):

"Moreover, with regard to inter-individual differences, mean Day 1 liking ratings for self-authored artworks positively covaried with activity in the right dorsal premotor/precentral cortex during creative reconstruction (own paintings > places; MNI: 58, 0, 50; z-score = 4.82; p_{FWER} = 0.023 at the voxel-level; see Fig 3b). There were also similar trends in the right anterior midcingulate (MNI:14, 22, 38; z-score = 3.7; puncorr = 0.0001 at the voxel-level) and in the right lingual gyrus (MNI: 22, -44, -2; z-score = 3.51; puncorr = 0.0002 at the voxel-level; see S5 Fig), though these effects did not survive correction for multiple comparisons at the whole brain level."

We accordingly added these results in the Discussion section (pages 29-30, lines 684-692), which now reads:

"Our data underscore the existence of a main creative-reconstruction network that distinguishes artistic-creation retrieval from familiar-place retrieval independently of appreciation. However, besides this left-sided system, a right-sided system was modulated by individual differences in the degree of appreciation of one's own art. Specifically, we found a positive relationship between the aesthetic value attributed to one's own

artworks and the recruitment of the right dorsal premotor cortex, accompanied by similar trends within the right anterior midcingulate and lingual cortices. These findings suggest that artists who assign higher aesthetic value to their own artworks may engage in the creative-reconstruction process by more strongly recruiting sensorimotor representations associated with the creative gesture, possibly extending to affective and visual-imagery components."

Minor Comments

8. Inaccurate claim regarding previous studies on expertise

(Lines 76–78) The statement that "all previous studies on aesthetic appreciation tried to bypass the effect of artistic education, including only participants naïve to the history or practice of visual art" is inaccurate as written. Several studies have specifically investigated the role of artistic expertise in aesthetic appreciation, including Leder et al. (2011, Psychology of Aesthetics, Creativity, and the Arts), Bimler et al. (2019, Acta Psychologica), and Darda & Cross (2022, Scientific Reports). At the neuroimaging level, Kirk et al. (2009, Brain and Cognition) investigated brain correlates of aesthetic expertise using fMRI. If the authors intend to restrict their claim specifically to fMRI studies on professional artists, they should say so explicitly. In either case, the statement should be moderated.

A.R. We thank the Reviewer for this correction. We agree that the previous wording was too broad and therefore inaccurate. Our intended claim was not that expertise has never been studied in aesthetic appreciation, but rather that previous fMRI work has rarely, if ever, investigated professional artists while they evaluate artworks and reconstruct their own creative process, especially within the same experimental design.

We have moderated the statement and clarified this point.

The revised Introduction now reads (page 4, lines 84-87): "Moreover, although previous behavioral experiments have investigated the role of artistic expertise on aesthetic appreciation [20-22], to our knowledge, there are no fMRI studies directly examining professional artists' responses to artworks and combining art viewing with the reconstruction of artistic creation in the same participants [16]."

9. Task instructions

(Lines 93 and 192) The authors describe asking participants to reconstruct "what they thought and felt when they conceived their own pieces of art." Were these the exact instructions given to participants? The precise wording of task instructions should be reported, as subtle differences in framing (e.g., emphasizing conception vs. physical execution) could meaningfully influence the cognitive and neural strategies employed.

A.R. We agree that the exact wording of the task instructions is important, especially because different framings could bias participants toward conceptual, emotional, or motor aspects of the creative process. We have now added precise instructions to the Methods section (pages 9-10, lines 216-218). The exact instructions were: "Imagine as vividly as possible what appeared in your mind and what you felt when you i) conceived the artwork that will be presented to you or ii) visited the place that will be presented to you for the first time". We note that the instruction did not explicitly ask participants to imagine physical execution, which makes the post-scanning reports of spontaneous motor imagery strategies particularly informative.

10. Attention-check question

(Lines 188 and 317–334) The random question used to maintain participants' attention during the aesthetic appreciation task is mentioned but not described in sufficient detail. What was the exact content of this question? How frequently was it presented? Critically, how was it modeled in the first-level GLM?

A.R. We thank the Reviewer for asking us to clarify this point. We have now expanded the Methods section (pages 10 and 15) to report the exact wording, frequency, timing, and first-level modelling of the attention-check question.

The exact question was: "Was this your own artwork or an artwork by another artist?" Participants were instructed to respond by pressing one button with the thumb if the artwork was their own and another button with the index finger if it had been made by another artist. Each mini-block included four artworks (two self-authored and two other-authored), one implicit baseline, and one attention-check question presented in random order. Thus, the question was presented once per mini-block, for a total of 10 times during the aesthetic judgment run.

The question remained on screen for up to 4 s. If participants responded before the end of this interval, a black screen was presented so that the total duration of the question-related event remained fixed at 5 s. In the first-level GLM, the question and the associated response were modelled as a regressor of no interest, with the onset corresponding to the onset of the question and the event duration set to 5 s.

11. Effect sizes

(Line 376) The authors report test statistics and p-values for all behavioral analyses, but effect sizes are not provided. In line with current best practices and guidelines, I recommend including appropriate effect size measures alongside each inferential test (for instance, Cohen's d for pairwise t-tests and partial/η^2p for ANOVA and linear mixed-effects models)

A.R. We thank the Reviewer for this suggestion. In the revised manuscript, we have now complemented the behavioral analyses with model estimates and 95% confidence intervals, in order to provide clearer information about the magnitude and precision of the observed effects. We preferred to report estimates and confidence intervals because they are more interpretable for mixed models.

12. Missing table for Figure 2b

While Tables 1 and 2 report the activations corresponding to Figures 2a and 2c respectively, no table appears to be provided for Figure 2b (lines 408–413), which reports the liking-related modulation within the aesthetic appreciation task. This should be added for completeness.

A.R. We thank the Reviewer for this suggestion. In the revised manuscript, the liking-related modulation originally shown in Figure 2b has been removed from Figure 2, which now presents only the study's main findings. Since these activations did not survive correction for multiple comparisons at the whole-brain level, they have been moved to the Supporting Information and are now reported for completeness in S5 Fig.

At the same time, we added a new main-text figure, Figure 3, specifically devoted to the liking-related analyses based on the aesthetic judgments collected on Day 1. Importantly, Figure 3 reports only the effects that survived correction for multiple comparisons, either after small volume correction within the independently defined field A1 for the aesthetic judgment task, or at the whole-brain level for the reconstruction by imagery task. All relevant cluster information, including MNI coordinates, z-scores, cluster size, and p-values, is reported in the Results section of the main text.

13. Missing reference to Figure S1

(Line 236) A reference to Figure S1 appears to be missing at this point in the text.

A.R. We thank the Reviewer for pointing this out. We have now added the missing reference to Figure S1 in the Procedure section of the revised manuscript.

14. Figure 1 — experimental design

The trial timeline figure would benefit from a clearer schematic of the overall experimental design, explicitly indicating the two contrasts of interest (own vs. others' paintings; recall of art creation vs. recall of places) and the number of stimuli per condition (if I understand correctly, 10 liked and 10 less-liked items per category).

A.R. We thank the Reviewer for this useful suggestion. We have revised the caption of Figure 1 to make the overall experimental design clearer. The caption now explicitly reports the two main contrasts of interest, namely self-authored versus other-authored paintings in the aesthetic judgment task (own > others), and reconstruction of art creation versus reconstruction of places in the imagery task (own paintings > places). It also specifies the number of stimuli included in each condition. Finally, we clarify that, within each stimulus category, the selected stimuli included 10 more-liked and 10 less-liked items based on the Day 1 ratings.

The caption now reads (page 11, lines 258-267): "Fig 1. Trial-timeline of the experimental tasks. The figure illustrates the trial-time line of the a) aesthetic judgment task, and the b) reconstruction by imagery task. In the aesthetic judgment task, artists viewed 20 self-authored and 20 other-authored paintings and mentally formulated an aesthetic judgment. The main contrast of interest was self-authored versus other-authored paintings. In the reconstruction by imagery task, artists mentally reconstructed either the moment in which they conceived one of their own artworks or the moment in which they first visited a now-familiar place. This task included 20 self-authored paintings and 20 familiar places, and the main contrast of interest was reconstruction of art creation versus reconstruction of places. For each stimulus category, the 20 selected items included 10 more-liked and 10 less-liked stimuli, based on the Day 1 ratings. Red frames indicate the onset of the events of interest in the fMRI analyses. For illustrative purposes, the figure includes two paintings by the artist Stefano Pizzi."

15. line 348

The phrase "liking had an effect on aesthetic appreciation" is potentially confusing. The authors presumably mean that aesthetic appreciation (liking) modulated neural responses?.

A.R. We agree that the previous wording was confusing. We have rephrased the sentence to state more precisely that we tested whether liking ratings collected on Day 1 modulated neural responses during the two tasks (i.e., aesthetic judgment task and reconstruction by imagery task; page 16, lines 384-386).

References

Van de Cruys, S., Frascaroli, J., & Friston, K. (2024). Order and change in art: towards an active inference account of aesthetic experience. *Philosophical Transactions of the Royal Society B: Biological Sciences*, *379*(1895). https://doi.org/10.1098/rstb.2022.0411

Ishizu, T., & Zeki, S. (2011). Toward a brain-based theory of beauty. PloS one, 6(7), e21852.
