# Supplementary material for: How artists experience their own art
Source: PLoS One. 2026 Aug 19;21(8):e0355737. doi: 10.1371/journal.pone.0355737 (PMC13489410; doi:10.1371/journal.pone.0355737)
Supplement: Peer review history

# Peer review: Decision Letter 0

**DOI:** 10.1371/journal.pone.0355737.r001

**Authors:** Federico Giove

25 May 2026

Dear Dr. Tomasetig,

Please submit your revised manuscript by Jul 09 2026 11:59PM. If you will need more time than this to complete your revisions, please reply to this message or contact the journal office at plosone@plos.org. When you're ready to submit your revision, log on to https://www.editorialmanager.com/pone/ and select the 'Submissions Needing Revision' folder to locate your manuscript file.

- A letter that responds to each point raised by the academic editor and reviewer(s). You should upload this letter as a separate file labeled 'Response to Reviewers'.
- A marked-up copy of your manuscript that highlights changes made to the original version. You should upload this as a separate file labeled 'Revised Manuscript with Track Changes'.
- An unmarked version of your revised paper without tracked changes. You should upload this as a separate file labeled 'Manuscript'.

If you would like to make changes to your financial disclosure, please include your updated statement in your cover letter. Guidelines for resubmitting your figure files are available below the reviewer comments at the end of this letter.

If applicable, we recommend that you deposit your laboratory protocols in protocols.io to enhance the reproducibility of your results. Protocols.io assigns your protocol its own identifier (DOI) so that it can be cited independently in the future. For instructions see: https://journals.plos.org/plosone/s/submission-guidelines#loc-laboratory-protocols. Additionally, PLOS ONE offers an option for publishing peer-reviewed Lab Protocol articles, which describe protocols hosted on protocols.io. Read more information on sharing protocols at https://plos.org/protocols?utm_medium=editorial-email&utm_source=authorletters&utm_campaign=protocols.

As the corresponding author, your ORCID iD is verified in the submission system and will appear in the published article. PLOS supports the use of ORCID, and we encourage all coauthors to register for an ORCID iD and use it as well. Please encourage your coauthors to verify their ORCID iD within the submission system before final acceptance, as unverified ORCID iDs will not appear in the published article. *Only*  the individual author can complete the verification step; PLOS staff *cannot*  verify ORCID iDs on behalf of authors.

We look forward to receiving your revised manuscript.

Kind regards,

Federico Giove, PhD

Academic Editor

PLOS One

Journal Requirements:

When submitting your revision, we need you to address these additional requirements.

1. Please ensure that your manuscript meets PLOS ONE's style requirements, including those for file naming. The PLOS ONE style templates can be found at

https://journals.plos.org/plosone/s/file?id=wjVg/PLOSOne_formatting_sample_main_body.pdf and

https://journals.plos.org/plosone/s/file?id=ba62/PLOSOne_formatting_sample_title_authors_affiliations.pdf

2. Please note that PLOS One has specific guidelines on code sharing for submissions in which author-generated code underpins the findings in the manuscript. In these cases, we expect all author-generated code to be made available without restrictions upon publication of the work. Please review our guidelines at https://journals.plos.org/plosone/s/materials-and-software-sharing#loc-sharing-code and ensure that your code is shared in a way that follows best practice and facilitates reproducibility and reuse.

3. When completing the data availability statement of the submission form, you indicated that you will make your data available on acceptance. We strongly recommend all authors decide on a data sharing plan before acceptance, as the process can be lengthy and hold up publication timelines. Please note that, though access restrictions are acceptable now, your entire data will need to be made freely accessible if your manuscript is accepted for publication. This policy applies to all data except where public deposition would breach compliance with the protocol approved by your research ethics board. If you are unable to adhere to our open data policy, please kindly revise your statement to explain your reasoning and we will seek the editor's input on an exemption. Please be assured that, once you have provided your new statement, the assessment of your exemption will not hold up the peer review process.

4. If the reviewer comments include a recommendation to cite specific previously published works, please review and evaluate these publications to determine whether they are relevant and should be cited. There is no requirement to cite these works unless the editor has indicated otherwise.

[Note: HTML markup is below. Please do not edit.]

Reviewers' comments:

Reviewer's Responses to Questions

**Comments to the Author**

1. Is the manuscript technically sound, and do the data support the conclusions?

Reviewer #1: Yes

Reviewer #2: Yes

**********

2. Has the statistical analysis been performed appropriately and rigorously?

Reviewer #1: Yes

Reviewer #2: Yes

**********

3. Have the authors made all data underlying the findings in their manuscript fully available?

The PLOS Data policy

Reviewer #1: Yes

Reviewer #2: Yes

**********

4. Is the manuscript presented in an intelligible fashion and written in standard English?

Reviewer #1: Yes

Reviewer #2: Yes

**********

Reviewer #1: This is an important study both for the questions asked and the methodology used, namely fMRI. The study investigated brain activity in professional artists with regards to their own artworks and those by others. The results are a contribution to the field of brain and art. The manuscript is well written in its present version and no special revisions are recommended.

Reviewer #2: This manuscript addresses an original and theoretically well-motivated research question, investigating the neurocognitive bases of aesthetic experience and artistic creation in professional painters by contrasting the Embodied Hypothesis with the Perceptual Learning and Inhibitory Hypothesis using fMRI. The experimental design is sophisticated, the stimulus preparation is careful and individualized, and the manuscript is clearly written. Overall, the work has solid scientific merit and makes a meaningful contribution to the emerging field of empirical neuroaesthetics. The revisions requested concern primarily the transparency in the presentation of certain results, a more balanced treatment of the two competing theoretical frameworks, and a more explicit discussion of the methodological limitations inherent to this line of research.

Major Comments

1. Theoretical framing of the hypotheses

I am not entirely convinced that the study hypotheses are formulated in a way that fully respects the theoretical frameworks they aim to test. Based on the original formulation of the Perceptual Learning and Inhibitory Hypothesis, I would expect greater motor inhibition and enhanced perceptual recruitment specifically for paintings that are aesthetically preferred, regardless of whether they are self-authored or made by others, since the theory is fundamentally grounded in the phenomenology of aesthetic pleasure, also with auditory stimuli (Sarasso, P., Barbieri, P., Del Fante, E., Bechis, L., Neppi-Modona, M., Sacco, K., & Ronga, I. (2022). Preferred music listening is associated with perceptual learning enhancement at the expense of self-focused attention. Psychonomic bulletin & review, 29(6), 2108-2121. ). Furthermore, I could not identify in the original theoretical sources a clear account of the creative process per se within this framework. I would therefore be cautious about advancing directional hypotheses in this regard. This does not diminish the value of the empirical findings: the results can certainly be discussed in light of this theoretical framework, but the authors should be more explicit about the assumptions and potential limitations involved in operationalizing these theories within the current experimental design.

3. Clusters not surviving correction for multiple comparisons

I agree that also non significant results has their value (Chen, G., Taylor, P. A., & Cox, R. W. (2017). Is the statistic value all we should care about in neuroimaging?. NeuroImage, 147, 952-959. ). However, several key results discussed in the Main Text and illustrated in Figure 2 do not reach significance after FWER correction. Although the authors flag these with footnotes, they receive equal narrative emphasis throughout the text and are displayed in the main figures without sufficiently prominent caveats. I recommend that the Discussion explicitly distinguish between statistically supported findings and exploratory trends, and that the main conclusions be anchored accordingly.

4. MRI data preprocessing

(Line 281) The data matrix was interpolated to produce voxels with 2 × 2 × 2 mm dimensions. What was the native acquisition? (4 × 3 × 3 mm?). It should be explicitly acknowledged that upsampling artificially estimates signal values between measured points and may inflate the apparent spatial extent of activation clusters. This limitation should be acknowledged in the Preprocessing section.

5. Aesthetic appreciation independently of authorship

(Line 501) A question that arises from the current design is whether the authors examined the neural correlates of aesthetic appreciation independently of authorship collapsing across self-authored and others' paintings to identify brain regions that track aesthetic liking regardless of who produced the artwork. It should also be noted that what was measured in the scanner during the appreciation task was an aesthetic judgment, not aesthetic appreciation per se. It would be informative to know whether neural responses differ as a function of liked versus less-liked paintings across both categories. In this light, I would suggest either modeling four regressors of interest (own liked, own less-liked, others' liked, others' less-liked) or, alternatively, correlating the Day 1 liking ratings with the beta estimates from the own > others contrast. What the current data most compellingly support is the statement at line 526 that observing their own paintings automatically triggers internal mental representations of the actions previously performed, while conclusion on aesthetic pleasure would be strengthened by the analyses suggested above.

6. Motor versus visual familiarity

(Lines 538–540 and 547–552) The authors draw on the Calvo-Merino studies to argue that differential motor activations for self-authored versus others' paintings reflect motor rather than visual familiarity. However, the claim at lines 538–540 that this inference "is supported by the distribution of activations" involves circular reasoning: the distribution of activations is precisely what needs to be explained, and invoking it as supporting evidence assumes the conclusion. The activation pattern is equally consistent with the interpretation that motor-like responses reflect motor familiarity specifically, which is in fact the most parsimonious account, but this cannot be dissociated from visual familiarity on the basis of the current data alone. The ideal design to address this question would have included art historians or art critics (i.e. individuals with extensive visual familiarity with the stimuli but no motor experience in producing them) to determine whether motor-like activations arise from visual familiarity alone or require prior motor engagement. Furthermore, the authors state at line 102 that they ensured a high degree of familiarity with others' paintings. It is unclear how this was formally assessed.

7. Discussion (Lines 563 ff. — Reconstruction by imagery task) In my view, this section presents the most compelling and original contribution of the entire manuscript. The finding that mental reconstruction of the creative process recruits a fronto-parietal network associated with motor cognition provides the most innovative evidence in favor of an embodied account of artistic experience, particularly with respect to creativity. I would encourage the authors to give this result greater prominence in the Abstract and conclusions. As a constructive suggestion, I recommend correlating the individual beta estimates from the regions identified in this contrast with the aesthetic liking ratings collected on Day 1. Such an analysis could reveal whether the degree of motor recruitment during creative reconstruction is modulated by how much the artists valued their own works aesthetically, a theoretically meaningful finding that would add an important individual-differences dimension to the current results.

Minor Comments

8. Inaccurate claim regarding previous studies on expertise

(Lines 76–78) The statement that "all previous studies on aesthetic appreciation tried to bypass the effect of artistic education, including only participants naïve to the history or practice of visual art" is inaccurate as written. Several studies have specifically investigated the role of artistic expertise in aesthetic appreciation, including Leder et al. (2011, Psychology of Aesthetics, Creativity, and the Arts), Bimler et al. (2019, Acta Psychologica), and Darda & Cross (2022, Scientific Reports). At the neuroimaging level, Kirk et al. (2009, Brain and Cognition) investigated brain correlates of aesthetic expertise using fMRI. If the authors intend to restrict their claim specifically to fMRI studies on professional artists, they should say so explicitly. In either case, the statement should be moderated.

9. Task instructions

(Lines 93 and 192) The authors describe asking participants to reconstruct "what they thought and felt when they conceived their own pieces of art." Were these the exact instructions given to participants? The precise wording of task instructions should be reported, as subtle differences in framing (e.g., emphasizing conception vs. physical execution) could meaningfully influence the cognitive and neural strategies employed.

10. Attention-check question

(Lines 188 and 317–334) The random question used to maintain participants' attention during the aesthetic appreciation task is mentioned but not described in sufficient detail. What was the exact content of this question? How frequently was it presented? Critically, how was it modeled in the first-level GLM?

11. Effect sizes

(Line 376) The authors report test statistics and p-values for all behavioral analyses, but effect sizes are not provided. In line with current best practices and guidelines, I recommend including appropriate effect size measures alongside each inferential test (for instance, Cohen's d for pairwise t-tests and partial/η²p for ANOVA and linear mixed-effects models)

12. Missing table for Figure 2b

While Tables 1 and 2 report the activations corresponding to Figures 2a and 2c respectively, no table appears to be provided for Figure 2b (lines 408–413), which reports the liking-related modulation within the aesthetic appreciation task. This should be added for completeness.

13. Missing reference to Figure S1

(Line 236) A reference to Figure S1 appears to be missing at this point in the text.

14. Figure 1 — experimental design

The trial timeline figure would benefit from a clearer schematic of the overall experimental design, explicitly indicating the two contrasts of interest (own vs. others' paintings; recall of art creation vs. recall of places) and the number of stimuli per condition (if I understand correctly, 10 liked and 10 less-liked items per category).

15. line 348

The phrase "liking had an effect on aesthetic appreciation" is potentially confusing. The authors presumably mean that aesthetic appreciation (liking) modulated neural responses?.

**********

what does this mean?). If published, this will include your full peer review and any attached files.

If you choose “no”, your identity will remain anonymous but your review may still be made public.

**Do you want your identity to be public for this peer review?**  For information about this choice, including consent withdrawal, please see our Privacy Policy

Reviewer #1: No

Reviewer #2: No

**********

[NOTE: If reviewer comments were submitted as an attachment file, they will be attached to this email and accessible via the submission site. Please log into your account, locate the manuscript record, and check for the action link "View Attachments". If this link does not appear, there are no attachment files.]

To ensure your figures meet our technical requirements, please review our figure guidelines: https://journals.plos.org/plosone/s/figures

You may also use PLOS’s free figure tool, NAAS, to help you prepare publication quality figures: https://journals.plos.org/plosone/s/figures#loc-tools-for-figure-preparation.

NAAS will assess whether your figures meet our technical requirements by comparing each figure against our figure specifications.

# Author comment: Author response to Decision Letter 1

**DOI:** 10.1371/journal.pone.0355737.r002

9 Jul 2026

To the Academic Editor Federico Giove

Subject: Revision of the Ms. PONE-D-25-42801 to PLOS ONE

Dear Editor Federico Giove,

Enclosed, please find the revised version of our manuscript ‘How artists experience their own art’, by G. Tomasetig & L.M. Sacheli, M.A. Musco, S. Pizzi, G. Basso, G.F. Spitoni, G. Bottini, L. Pizzamiglio, E. Paulesu.

We would like to thank you for giving us the opportunity to submit a revised version of our manuscript. We are grateful to the Reviewers for their useful comments that we took into the highest consideration. We clarified the critical points raised and, having done so, we believe that the manuscript is now improved.

In the following point-by-point response letter, the Reviewers’ comments are reported in black bold font; our replies are in blue and preceded by "A.R." (Authors Response). Changes to the main text are highlighted in yellow.

We hope that you and the Reviewers will be satisfied with the revision and that the paper can be accepted for publication in PLOS ONE.

If there is anything else you may expect from our side, please do not hesitate to ask at any time.

We look forward to hearing from you.

With kindest regards,

Giulia Tomasetig, Lucia Maria Sacheli, Eraldo Paulesu and Co-Authors

Email: g.tomasetig@campus.unimib.it, lucia.sacheli@unimib.it, eraldo.paulesu@unimib.it

Journal Requirements

1. Please ensure that your manuscript meets PLOS ONE's style requirements, including those for file naming. The PLOS ONE style templates can be found at

https://journals.plos.org/plosone/s/file?id=wjVg/PLOSOne_formatting_sample_main_body.pdf and

https://journals.plos.org/plosone/s/file?id=ba62/PLOSOne_formatting_sample_title_authors_affiliations.pdf

A.R. We thank the Editorial Office for this reminder. We have revised the manuscript to comply with PLOS ONE style requirements. In particular, we have moved the References before the Supporting Information section and checked the structure, file naming, and formatting of the revised submission against the journal templates.

2. Please note that PLOS One has specific guidelines on code sharing for submissions in which author-generated code underpins the findings in the manuscript. In these cases, we expect all author-generated code to be made available without restrictions upon publication of the work. Please review our guidelines at https://journals.plos.org/plosone/s/materials-and-software-sharing#loc-sharing-code and ensure that your code is shared in a way that follows best practice and facilitates reproducibility and reuse.

A.R. We confirm that no author-generated code underpins the findings reported in the manuscript. The analyses were conducted using standard, publicly available software packages described in the Methods section (SPM12 for fMRI preprocessing and analyses; Jamovi/GAMLj for behavioral analyses). Accordingly, there is no custom code to be shared.

3. When completing the data availability statement of the submission form, you indicated that you will make your data available on acceptance. We strongly recommend all authors decide on a data sharing plan before acceptance, as the process can be lengthy and hold up publication timelines. Please note that, though access restrictions are acceptable now, your entire data will need to be made freely accessible if your manuscript is accepted for publication. This policy applies to all data except where public deposition would breach compliance with the protocol approved by your research ethics board. If you are unable to adhere to our open data policy, please kindly revise your statement to explain your reasoning and we will seek the editor's input on an exemption. Please be assured that, once you have provided your new statement, the assessment of your exemption will not hold up the peer review process.

A.R. We confirm that our behavioral and neuroimaging data have already been deposited in the Open Science Framework (OSF) and are currently available for peer review through the following view-only link: https://osf.io/e4fdb/?view_only=7f3dbfdfff70480fb982670c994c01e8. Should the manuscript be accepted, we will make the repository publicly accessible at https://osf.io/e4fdb/.

4. If the reviewer comments include a recommendation to cite specific previously published works, please review and evaluate these publications to determine whether they are relevant and should be cited. There is no requirement to cite these works unless the editor has indicated otherwise.

A.R. We have reviewed and evaluated the publications suggested by the Reviewer. Although we understand that there was no requirement to cite them, we found some of these references useful for providing a more complete account of previous work on artistic expertise. Accordingly, we have incorporated the relevant references in the revised manuscript where appropriate.

Reviewer:1

Reviewer #1: This is an important study both for the questions asked and the methodology used, namely fMRI. The study investigated brain activity in professional artists with regards to their own artworks and those by others. The results are a contribution to the field of brain and art. The manuscript is well written in its present version and no special revisions are recommended.

A.R. We sincerely thank the Reviewer for their positive evaluation of our work and for recognizing the contribution of the study to the field of cognitive neuroscience of art.

Reviewer:2

Reviewer #2: This manuscript addresses an original and theoretically well-motivated research question, investigating the neurocognitive bases of aesthetic experience and artistic creation in professional painters by contrasting the Embodied Hypothesis with the Perceptual Learning and Inhibitory Hypothesis using fMRI. The experimental design is sophisticated, the stimulus preparation is careful and individualized, and the manuscript is clearly written. Overall, the work has solid scientific merit and makes a meaningful contribution to the emerging field of empirical neuroaesthetics. The revisions requested concern primarily the transparency in the presentation of certain results, a more balanced treatment of the two competing theoretical frameworks, and a more explicit discussion of the methodological limitations inherent to this line of research.

A.R. We thank the Reviewer for their thoughtful and constructive assessment of our manuscript and for recognizing the potential contribution of the study. We have carefully considered the Reviewer’s comments, which helped us improve the manuscript considerably. Below, we address each point in detail.

Major Comments

1. Theoretical framing of the hypotheses

I am not entirely convinced that the study hypotheses are formulated in a way that fully respects the theoretical frameworks they aim to test. Based on the original formulation of the Perceptual Learning and Inhibitory Hypothesis, I would expect greater motor inhibition and enhanced perceptual recruitment specifically for paintings that are aesthetically preferred, regardless of whether they are self-authored or made by others, since the theory is fundamentally grounded in the phenomenology of aesthetic pleasure, also with auditory stimuli (Sarasso, P., Barbieri, P., Del Fante, E., Bechis, L., Neppi-Modona, M., Sacco, K., & Ronga, I. (2022). Preferred music listening is associated with perceptual learning enhancement at the expense of self-focused attention. Psychonomic bulletin & review, 29(6), 2108-2121. ).

A.R. We thank the Reviewer for this important clarification. We agree that the theoretical framework on aesthetic perception that we refer to most directly predicts enhanced perceptual processing and motor inhibition for aesthetically preferred stimuli, regardless of whether they are self-authored or produced by another artist. We also agree that this point should have been made more explicit in the previous version of the manuscript. Accordingly, in the revised manuscript, we now distinguish more clearly between two levels of the argument.

First, we describe the canonical prediction of the Perceptual Learning and Inhibitory Hypothesis in the domain of aesthetic perception, namely that aesthetic pleasure and preferred stimuli should be associated with enhanced perceptual processing and motor inhibition.

Second, we clarify that our prediction regarding self-authored versus other-authored artworks derived from the broader predictive-coding framework within which this hypothesis, at least in the aesthetic perception domain, is situated. More specifically, our reasoning was grounded in predictive-coding and active-inference accounts of aesthetic experience, according to which aesthetic pleasure is closely linked to uncertainty reduction, epistemic value, sense-making, and the progressive construction of a generative model of the artwork (i.e., a model of how the stimulus was generated, of what feelings and intentions occupied the artist’s mind when realizing the brushstrokes on the canvas; Van de Cruys, Frascaroli & Friston, 2024). From this perspective, self-authored works may entail lower epistemic uncertainty for the artist (and, consequently, fewer opportunities for learning) because the artist has privileged access to the generative process that produced the work. By contrast, works produced by others may offer greater opportunity for prediction updating and epistemic exploration. We now present this reasoning more explicitly as an inference derived from the broader predictive-coding framework, while also acknowledging that it goes beyond the original theoretical framework's most direct prediction.

In the revised manuscript, we have added/modified the text as follows (pages 5-6, lines 111-134):

“Comparing artists’ responses to their own versus others’ paintings allowed us to investigate whether the authorship factor modulates the neurofunctional responses during the aesthetic judgment task through embodied simulation mechanisms or through perceptual learning and inhibitory processes. The Embodied Hypothesis provides a relatively direct prediction for the self/other contrast. People can recognize and discriminate the products of their past actions from those generated by others [23,24]. This so-called “authorship effect” may be driven by the perception of self-kinematic motor information embedded in static traces, rather than by visual familiarity alone [25,26] or episodic memory [27]. Accordingly, the motor system may resonate more strongly with signals stemming from prior self-produced actions [24,28]. From this perspective, self-generated paintings should elicit motor representations of gestures that artists previously enacted during their creation. Thus, professional artists should show stronger recruitment of brain regions involved in motor resonance while observing their own vs. others’ paintings.

By contrast, the Perceptual Learning and Inhibitory Hypothesis was originally formulated to explain aesthetic pleasure as emerging from perceptual learning, uncertainty reduction, and “insight” (i.e., Aha experience) on the generative model of the artwork (i.e., a model of the artwork's life cycle, from its conception to its generation [13]). The general prediction from this model is an enhanced perceptual processing and motor inhibition for aesthetically preferred stimuli. However, using the same model, one can make predictions on a self/other authorship manipulation of the stimuli as the one adopted in this experiment. Other-authored artworks may retain greater epistemic uncertainty than self-authored ones, and therefore greater potential for prediction-updating. By contrast, artists have more precise predictions about their own works because they know the actions and intentions that gave rise to them. Consistent with evidence for enhanced prediction and sensory attenuation for self-generated actions [23,28–30], viewing others’ paintings should therefore engage perceptual and motor-inhibitory brain regions more strongly, whereas viewing one’s own paintings should place lower demands on perceptual learning. To summarize, what counts here is that the two theories make opposite functional anatomical predictions about the authorship effects”.

Furthermore, I could not identify in the original theoretical sources a clear account of the creative process per se within this framework. I would therefore be cautious about advancing directional hypotheses in this regard. This does not diminish the value of the empirical findings: the results can certainly be discussed in light of this theoretical framework, but the authors should be more explicit about the assumptions and potential limitations involved in operationalising these theories within the current experimental design.

A.R. We agree with the Reviewer that the theoretical sources related to aesthetic perception and predictive coding do not provide, by themselves, a direct account of the creative process per se. We have therefore revised the manuscript to make the assumptions underlying our theoretical framing more explicit. In our manuscript, the term Perceptual Learning and Inhibitory Hypothesis is used to refer to a broader “Apollonian” position that brings together two distinct but related lines of research: predictive-coding accounts of aesthetic experience, on the one hand, and creative-cognition accounts of artistic production, on the other. These two literatures do not fully belong to the same theoretical framework. In particular, while the literature on aesthetic perception can be situated within predictive-coding and active-inference accounts, the creative-cognition literature does not directly fall within the Predictive Coding framework. However, we considered them together because both emphasize the role of inhibitory processes, although in different domains and with different functional meanings. We have therefore clarified that our theoretical framing does not imply that the creative-cognition literature is part of the Predictive Coding framework. Rather, our proposal reflects a broader attempt to relate perceptual and productive aspects of art experience within the same theoretical space.

The revised Introduction now reads (page 4, lines 77-82):

“Although such Creative Cognition literature does not directly fall within the Predictive Coding framework, it is relevant to the present theoretical contrast because it also emphasizes the role of inhibitory processes, which would be crucial to creating something new. Within this line and following a previous attempt to situate perceptual and productive aspects of art experience within a single theoretical space ([16] see also [17,18]), we refer to this broader “Apollonian” position as the Perceptual Learning and Inhibitory Hypothesis.”

We have revised the relevant passage as follows (pages 6-7, lines 135-144):

“The two frameworks also differ in their predictions for the reconstruction by imagery task. On the one hand, according to the Embodied Hypothesis, during the reconstruction of creative moments, professional artists should recruit brain regions involved in sensorimotor simulation to a greater extent than in the control condition. On the other hand, according to the Perceptual Learning and Inhibitory Hypothesis, inhibitory-control processes should play a relevant role in the reconstruction of creative moments. This prediction is grounded in the Creative Cognition literature, where inhibitory control has been proposed to support the suppression of dominant or conventional associations in order to generate novel solutions [14,15]. Thus, in the present study, we used the two tasks to examine whether artists’ aesthetic judgment and reconstruction of the creative moment were more strongly characterized by sensorimotor/motor-simulation processes or by perceptual-learning and inhibitory-control mechanisms”.

3. Clusters not surviving correction for multiple comparisons

I agree that also non significant results has their value (Chen, G., Taylor, P. A., & Cox, R. W. (2017). Is the statistic value all we should care about in neuroimaging?. NeuroImage, 147, 952-959. ). However, several key results discussed in the Main Text and illustrated in Figure 2 do not reach significance after FWER correction. Although the authors flag these with footnotes

Attachment Submitted filename: Tomasetig,Sacheli_Cover&PointByPoint.pdf

# Peer review: Decision Letter 1

**DOI:** 10.1371/journal.pone.0355737.r003

**Authors:** Federico Giove

26 Jul 2026

How artists experience their own art

PONE-D-25-42801R1

Dear Dr. Tomasetig,

We’re pleased to inform you that your manuscript has been judged scientifically suitable for publication and will be formally accepted for publication once it meets all outstanding technical requirements.

Within one week, you’ll receive an e-mail detailing the required amendments. When these have been addressed, you’ll receive a formal acceptance letter and your manuscript will be scheduled for publication.

An invoice will be generated when your article is formally accepted. Please note, if your institution has a publishing partnership with PLOS and your article meets the relevant criteria, all or part of your publication costs will be covered. Please make sure your user information is up-to-date by logging into Editorial Manager at Editorial Manager® and clicking the ‘Update My Information' link at the top of the page. For questions related to billing, please contact billing support.

If your institution or institutions have a press office, please notify them about your upcoming paper to help maximize its impact. If they’ll be preparing press materials, please inform our press team as soon as possible -- no later than 48 hours after receiving the formal acceptance. Your manuscript will remain under strict press embargo until 2 pm Eastern Time on the date of publication. For more information, please contact onepress@plos.org.

Kind regards,

Federico Giove, PhD

Academic Editor

PLOS One

Additional Editor Comments (optional):

Reviewers' comments:

Reviewer's Responses to Questions

**Comments to the Author**

Reviewer #2: All comments have been addressed

**********

2. Is the manuscript technically sound, and do the data support the conclusions?

Reviewer #2: Yes

**********

3. Has the statistical analysis been performed appropriately and rigorously?

Reviewer #2: Yes

**********

4. Have the authors made all data underlying the findings in their manuscript fully available?

The PLOS Data policy

Reviewer #2: Yes

**********

5. Is the manuscript presented in an intelligible fashion and written in standard English?

Reviewer #2: Yes

**********

Reviewer #2: I thank the authors for their careful consideration of my comments. I hope that the revisions have improved the manuscript and that my suggestions have contributed constructively to the discussion and overall quality of the work.

**********

what does this mean?). If published, this will include your full peer review and any attached files.

If you choose “no”, your identity will remain anonymous but your review may still be made public.

**Do you want your identity to be public for this peer review?**  For information about this choice, including consent withdrawal, please see our Privacy Policy

Reviewer #2: No

**********

# Editor report: Acceptance letter

**DOI:** 10.1371/journal.pone.0355737.r004

**Authors:** Federico Giove

PONE-D-25-42801R1

PLOS One

Dear Dr. Tomasetig,

I'm pleased to inform you that your manuscript has been deemed suitable for publication in PLOS One. Congratulations! Your manuscript is now being handed over to our production team.

At this stage, our production department will prepare your paper for publication. This includes ensuring the following:

* All references, tables, and figures are properly cited

* All relevant supporting information is included in the manuscript submission,

* There are no issues that prevent the paper from being properly typeset

You will receive further instructions from the production team, including instructions on how to review your proof when it is ready. Please keep in mind that we are working through a large volume of accepted articles, so please give us a few days to review your paper and let you know the next and final steps.

Lastly, if your institution or institutions have a press office, please let them know about your upcoming paper now to help maximize its impact. If they'll be preparing press materials, please inform our press team within the next 48 hours. Your manuscript will remain under strict press embargo until 2 pm Eastern Time on the date of publication. For more information, please contact onepress@plos.org.

You will receive an invoice from PLOS for your publication fee after your manuscript has reached the completed accept phase. If you receive an email requesting payment before acceptance or for any other service, this may be a phishing scheme. Learn how to identify phishing emails and protect your accounts at https://explore.plos.org/phishing.

If we can help with anything else, please email us at customercare@plos.org.

Thank you for submitting your work to PLOS ONE and supporting open access.

Kind regards,

PLOS ONE Editorial Office Staff

on behalf of

Dr. Federico Giove

Academic Editor

PLOS One
